# Defense against adversarial attacks: robust and efficient compressed optimized neural networks

**DOI:** 10.1038/s41598-024-56259-z

**Published:** 2024-03-17

**Authors:** Insaf Kraidia, Afifa Ghenai, Samir Brahim Belhaouari

**Affiliations:** 1 LIRE Laboratory, University of Constantine 2 - Abdelhamid Mehri, Ali Mendjeli Campus, 25000 Constantine, Algeria; 2 Division of Information and Computing Technology, College of Science and Engineering, Hamad Bin Khalifa University, Ar-Rayyan, Qatar

**Keywords:** Adversarial attacks, Generative pre-trained transformer (GPT), Compression, Multi expert, Mathematics and computing, Computer science

## Abstract

In the ongoing battle against adversarial attacks, adopting a suitable strategy to enhance model efficiency, bolster resistance to adversarial threats, and ensure practical deployment is crucial. To achieve this goal, a novel four-component methodology is introduced. First, introducing a pioneering batch-cumulative approach, the exponential particle swarm optimization (ExPSO) algorithm was developed for meticulous parameter fine-tuning within each batch. A cumulative updating loss function was employed for overall optimization, demonstrating remarkable superiority over traditional optimization techniques. Second, weight compression is applied to streamline the deep neural network (DNN) parameters, boosting the storage efficiency and accelerating inference. It also introduces complexity to deter potential attackers, enhancing model accuracy in adversarial settings. This study compresses the generative pre-trained transformer (GPT) by 65%, saving time and memory without causing performance loss. Compared to state-of-the-art methods, the proposed method achieves the lowest perplexity (14.28), the highest accuracy (93.72%), and an 8 × speedup in the central processing unit. The integration of the preceding two components involves the simultaneous training of multiple versions of the compressed GPT. This training occurs across various compression rates and different segments of a dataset and is ultimately associated with a novel multi-expert architecture. This enhancement significantly fortifies the model's resistance to adversarial attacks by introducing complexity into attackers' attempts to anticipate the model's prediction integration process. Consequently, this leads to a remarkable average performance improvement of 25% across 14 different attack scenarios and various datasets, surpassing the capabilities of current state-of-the-art methods.

## Introduction

In the ever-evolving landscape of cybersecurity, where digital communication plays a pivotal role, adversarial attacks have expanded to encompass traditional vectors with more insidious and sophisticated methods. One such intriguing facet is the realm of adversarial black box text attacks, in which the attacker lacks access to the internal workings or parameters of the target neural network (NN) models^1^. Defending against black-box attacks poses a formidable challenge, primarily due to defenders' limited access to the inner parameters of the target NN model^2^. Nonetheless, researchers have proposed various defense approaches to improve the robustness of natural language processing (NLP) models and mitigate the impact of attacks. Some of these defense techniques include adversarial training^3^, which incorporates adversarial examples into the training data; defensive distillation^4^, which adds an extra layer of protection by training a different model; input transformation^5^, which uses random resizing, rotations, and crops; and ensemble methods^6^, which combine multiple models.

However, these defense approaches have their drawbacks. Strengthening defense approaches can sometimes reduce the accuracy of clean data and may not generalize well to unseen or sophisticated attacks^7^. Additionally, they often necessitate additional computations during training or inference, which can significantly increase computational complexity^8^, leading to longer training times and slower real-time deployment^9^. Specifically, approaches such as ensemble methods or defensive distillation can require the storage of multiple models or intermediate data, which increases memory usage^10,11^. Consequently, we are searching for adversarial attack protection techniques that enhance NN models and enable the smooth integration of machine learning models into real-world applications.

**Figure 1:**
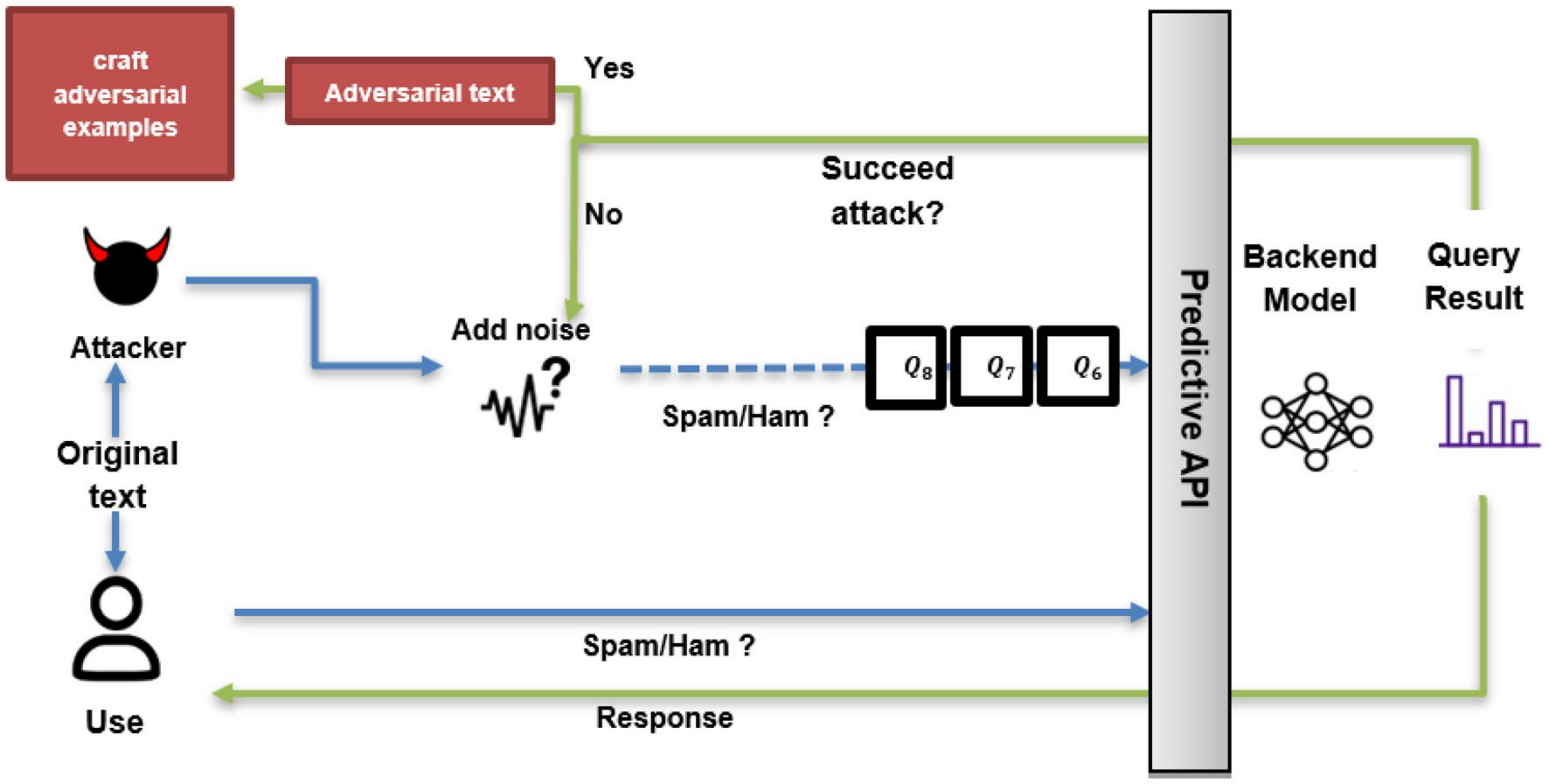
The process of black-box adversarial attacks. Attackers assemble a dataset of input texts and their correct labels from the target model. Subsequently, they iteratively submit these texts to the black-box model, observe outputs, and generate adversarial examples. Techniques such as genetic algorithms, transfer-based attacks, or optimization algorithms are often employed to craft adversarial examples that cause misclassifications.

As shown in Fig. 1, attackers often query the target model repeatedly to acquire the necessary information for optimizing their strategy. Many attacks rely on a searching algorithm (e.g., greedy or genetic) to iteratively replace each character/word in a sentence with a perturbation candidate to optimize the choice of characters/words and how they should be crafted to attack the target model. Even though this process is effective in terms of attack performance, they assume that the model’s parameters remain “unchanged” and that the model outputs “coherent” signals during the iterative search. However, our essential intuition is to obfuscate attackers by violating this assumption. Specifically, we want to develop an algorithm that automatically utilizes diverse models during inference. This can be done by training a stochastic multi-expert approach enhancing the deep neural network model, particularly the generative pre-trained transformer, by exclusively modifying its final layer. This modification transforms the model into an ensemble of multiple expert predictors associated with stochastic weights^12^.

However, despite the advantage of enhanced accuracy, this method may not fully capture the diversity of predictions. When consistently selecting the single best performing architecture, there is a potential risk that the model may not sufficiently diversify its predictions^13^. A limited range of model outputs could restrain its ability to uncover new insights or handle unexpected data patterns. To address these challenges, we developed an innovative adversarial defense methodology. Our methodology introduces a stochastic multi-expert approach, combining the outputs of expert architectures within each head and averaging them to generate more balanced and robust predictions. To enhance the ability of the model to generalize across diverse scenarios and data distributions in an adversarial environment, we create multiple model variants instead of depending on a single base model. To obfuscate the attackers more, base models employ distinct compression settings and undergo training on randomly selected and diverse portions of the dataset. Adopting a random selection method among the base model versions for making final predictions introduces an element of unpredictability into the model's decision-making process, making it more challenging for adversaries to craft effective adversarial examples.

Concerning the temporal and memory constraints of defensive models, researchers have focused their efforts on compressing the PLMs within textual defense models^14–16^. Most of the existing research in this domain has focused primarily on optimizing encoder-based or decoder-based models, such as QuantGPT^17^, KnGPT^18^, and SparseGPT^19^. However, certain compression techniques are tailored to specific pre-trained language model architectures. For instance, the KnGPT technique is explicitly designed for GPT-2. Applying this approach to other GPT versions or models does not yield the same benefits. On the other hand, some techniques, such as QuantGPT, provide compression methods that efficiently reduce computational resource requirements but impact the model's performance and generality. Conversely, techniques such as KnGPT and SparseGPT do not affect performance but require significant computational resources for the compression process. To address resource challenges, we present a novel smart compression approach to alleviate computational demands, encompassing decoder and encoder-based PLMs and DNNs. Furthermore, we propose a novel batch approach that integrates ExPSO to consolidate the model performance during the compression process. This integration accelerates convergence and mitigates the risk of encountering local optima. Our contributions can be summarized as follows:

- We propose a new approach for DNNs that efficiently defends against 14 adversarial attacks and assesses its effectiveness using three public datasets.
- We introduce a novel compression approach designed for differentiable decoder-based and encoder-based pre-trained language models (PLMs) and assess its effectiveness using GPT.
- To the best of our knowledge, the research presented in this paper is the first work to integrate ExPSO with a compression technique to optimize DNN parameters.
- We develop a novel, effective batch approach for enhancing DNN performance.

The rest of the paper is organized as follows. We provide related work in the “Related work” section. We present our methodology in “The proposed methodology” section. We describe the experiments, results, and discussion in the “Experimental evaluation” section. Finally, we conclude in the “Conclusion” section.

## Related work

In the Blackbox attack, the generated adversarial examples can be categorized into three major types based on the level at which the attacker manipulates the input text^20^: character-level attacks, which manipulate individual characters or introduce new examples for model confusion; word-level attacks, which perturb individual words in the text using synonyms or antonyms to alter the text's sentiment and meaning; and sentence-level attacks, which involve rearranging word order, adding irrelevant information, negating original statements, or reversing their intended meaning^21^. Table 1 provides more detailed information on the attack models involved in the OpenAttack framework^22^ and outlines their main concepts. Moreover, Table 2 illustrates how each adversarial text attack can manipulate text inputs to deceive NLP models.

**Table 1:** Adversarial text attack methods and their main ideas across different levels.

Attack level	Attack	Main idea
Character-level	Generating Adversarial Text Against Real-world Applications (TextBugger)^23^	Greedy word substitution and character manipulation
	Universal Adversarial Triggers for Attacking and Analyzing NLP (UAT)^1^	Gradient-based word or character manipulation
	Visually Attacking and Shielding NLP Systems (VIPER) ^24^	Visually similar character substitution
	Black-box Generation of Adversarial Text Sequences to Evade Deep Learning Classifiers (DeepWordBug) ^25^	Greedy character manipulation
	TextFooler (TF)^26^	Greedy word substitution
Word-level	White-Box Adversarial Examples for Text Classification (HotFlip)^27^	Gradient-based word or character substitution
	Generating Natural Language Adversarial Examples through Probability Weighted Word Saliency (PWWS)^28^	Greedy word substitution
	Generating Natural Language Adversarial Examples (Genetic)^29^	Genetic algorithm-based word substitution
	Word-level Textual Adversarial Attacking as Combinatorial Optimization (SememePSO)^30^	Particle swarm optimization-based word substitution
	Adversarial Attack Against BERT Using BERT (BERT-ATTACK)^31^	Greedy contextualized word substitution
	BERT-based Adversarial Examples for Text Classification (BAE)^32^	Greedy contextualized word substitution and insertion
	Semantically Equivalent Adversarial Rules for Debugging NLP Models (SEA)^33^	Rule-based paraphrasing
Sentence-level	Adversarial Example Generation with Syntactically Controlled Paraphrase Networks (SCPN)^34^	Paraphrasing
	Generating Natural Adversarial Examples (GAN)^35^	Text generation by encoder–decoder

**Table 2:** Adversarial text attack levels and corresponding text modification examples.

Adversarial text attack level	Original text	Modified text
Character-level	"The qu**i**ck b**r**own fox jumps ov**e**r the l**a**zy d**o**g."	"The qu**x**ck b**!**own fox jumps ov**3**r the 1**4**zy d**@**g."
Word-level	"The movie was **really** good."	"The movie was **quite** bad."
Sentence-level	"Climate change **is a global** concern. **We must take action** to reduce carbon emissions."	"Climate change **isn't a** concern. **There's no need to** reduce carbon emissions."

Significant values are in bold and underline.

In the realm of defending against adversarial attacks, different techniques have introduced a spectrum of enhancements. A variation of the ensemble baseline known as diversity training (DT) was created, in which a regularization component was added to enhance the alignment of gradient vectors among sub-models and encourage a wider range of expertise^4^. By emphasizing the diversification of each sub-model competence at the class level inside the ensemble baseline, adaptive variety promotion (ADP) further contributed to the enhancement of variety, particularly in nonmaximal predictions^6^. Conversely, mixup training aims to increase the model's flexibility in response to linear transformations between continuous embeddings of the training data^36^. To maximize the negative log-likelihood loss on both the initial training data and adversarial inputs, adversarial training (AdvT) uses semi-supervised learning, which improves model robustness^37^. To ensure the model's resilience against adversarial perturbations and misspelling in the input text, a robust word recognizer (ScRNN) was implemented as a preprocessing step before the base model made predictions^38^. Finally, to generate a stochastic weighted ensemble, the SHIELD adopts a novel strategy by altering and retraining the last layer of a textural neural network^12^.

In defending against adversarial attacks, each prior study has contributed substantially to addressing specific challenges; however, certain limitations persist. Both DT and ADP concentrate on diminishing the dimensionality of the adversarial subspace and promoting transferability. Nevertheless, these methods necessitate training multiple diverse sub-models or ensembles, incurring computational and resource-intensive demands. Furthermore, they compel adversaries to simultaneously target a predetermined ensemble of diverse sub-models, potentially limiting adaptability to evolving attack strategies and failing to cover the full spectrum of potential adversarial scenarios. On the other hand, AdvT, ScRNN (semi-character-based RNN), and Mixup adopt relatively straightforward training processes that may not thoroughly explore the complete range of potential adversarial perturbations or attack strategies^39^. While the SHIELD aims to prevent model specialization and enhance generalization, it falls short of fully mitigating adversarial inputs. This approach may restrict insights due to its reliance on a single best-performing architecture. These considerations highlight the need for further advancements in adversarial attack defense to overcome existing limitations and create more robust and adaptable solutions.

In contrast to existing approaches, our innovative methodology employs a multifaceted approach to address prevailing challenges. By placing a greater emphasis on diluting direct attacks rather than focusing solely on transferability, we strategically prompt adversaries to target stochastic sub-model variations during every inference pass^21^. This intentional diversification mitigates the challenges traditionally posed by diversity training and adaptive variety promotion, providing a robust defense strategy. The nuanced focus is on disrupting direct attacks and introducing stochastic elements into the inference process (e.g., heads expert's learnable params.) sets our approach apart, offering a more adaptive and effective defense mechanism against adversarial threats^25,40^. We present a more stochastic approach to address the constraints posed by AdvT, ScRNN, and Mixup, heightening the difficulty of adversarial attacks. First, our solution incorporates a multi-version training method employing various base model versions, each of which undergoes compression with distinct values. This multi-version training allows the model to learn from equivalent yet diverse perspectives^41^. Leveraging random selection further enhances the stochasticity of the model's internal structure^42^, making it more challenging for potential attackers to discern the network's intricacies (increased unpredictability). Second, our solution incorporates a multi-expert architecture based on Gumbel noise, which has been shown to act as a regularizer, preventing models from over-relying on specific features^43^. Unlike the SHIELD, our stochastic system emphasizes utilizing an average-performing architecture instead of a single best-performing architecture. This strategic choice enables the model to integrate insights from various architectures, encompassing different facets of the data. Doing so enhances the model's generalization capabilities, allowing it to capture a broader spectrum of patterns and features^44,45^.

Researchers have proposed several techniques for dealing with memory and temporal limitations in defensive models, including Adam and Particle Swarm Optimization variants. Table 3 presents a comprehensive comparison study that clarifies the distinctive features and uses of different optimization strategies. However, due to the distinctive qualities of language datasets and inputs, the generic nature of these methodologies becomes inadequate when working with pre-trained language models^46^. Moreover, PLMs require particular techniques that consider the massive datasets on which they are trained and the sequential nature of language, necessitating the development of specialized optimization algorithms^47^.

**Table 3:** Optimization techniques: a detailed comparative analysis.

Optimization method	Description
Adagrad (Adaptive Gradient Algorithm)^48^	Adapts learning rates for each parameter based on historical gradient information
Adam (Adaptive Moment Estimation)^49^	Combines advantages of Adagrad and Root Mean Squared Propagation (RMSprop) and adapts the learning rates individually for each parameter
Adadelta^50^	Extension of Adagrad addressing diminishing learning rate
ADAPLUS^51^	Integrates Nesterov momentum and precise step size adjustment on an AdamW basis
Adan^52^	Adaptive Nesterov momentum algorithm for optimizing deep models faster
Phasor Particle Swarm Optimization (PPSO)^53^	Replaces control parameters with a scalar phasor angle based on trigonometric functions
Fitness-based Multirole PSO (FMPSO)^54^	Integrates a sub-social learning part into standard PSO to enhance search mechanisms
Multi-Swarm PSO (MSPSO)^55^	Utilizes dynamic strategies to divide swarms, regroup them, and avoid local minima based on historical information
Expanded PSO (XPSO)^56^	Integrates forgetting ability and multi-exemplar concept into standard PSO for improved optimization

In recent research endeavors, innovative techniques have been introduced to address the challenges posed by PLMs. Tao et al.^17^ present QuantGPT, a technique incorporating token-level contrastive distillation and module-wise dynamic scaling and demonstrates significant improvements over existing compression methods. Edalati et al.^18^ introduced another approach known as KnGPT2, which employs Kronecker decomposition to compress linear transformations within the GPT-2 model. This mathematical method breaks down intricate matrices into simpler components, reducing computational demands while preserving representational capacity. Song et al.^19^ propose LightPAFF, a two-stage knowledge distillation method enabling the transmission of knowledge from a larger teacher model to a more compact student model, empowering the lightweight model to retain comparable accuracy. On the other hand, SparseGPT introduces a one-shot pruning strategy, framing pruning as an extensive sparse regression problem solved using an approximate sparse regression solver, thereby eliminating the need for retraining^57^. While recent advancements have been made to address computational demands and eliminate the need for retraining, challenges persist with current methods. KnGPT2, for instance, exhibits limited applicability to specific GPT versions. On the other hand, QuantGPT introduces performance and generality concerns, and both SparseGPT and LightPAFF demand considerable computational resources during compression^58^.

Our compression technique is designed to operate autonomously from the internal model architecture. Its design hinges on taking the objective function as input, encompassing the model structure, training, and evaluation functions. This level of flexibility enables the technique to adapt seamlessly to various model structures and dimensions, contrasting KnGPT2, which is tailored explicitly for a predetermined architecture. We have incorporated a batch portion strategy to alleviate the need for extensive computational resources in the compression process. This approach involves utilizing a fraction of the overall data in each compression iteration. By adopting this strategy, we optimize efficiency, allowing particles to identify the optimal global position without imposing excessive computational demands^59^. Traditional compression methods for neural network models often raise concerns about their potential negative impact on model robustness^3,14,15,17,19^. In our approach, we avoid directly reducing or compressing the model, as such actions have been associated with detrimental effects on model parameters. Instead, our technique adopts a two-fold strategy. First, we embrace a gradual compression approach, progressively applying different compression percentages while closely monitoring the model's accuracy. This step-by-step compression allows us to assess the impact on model performance before advancing further with the compression process. Second, we leverage an optimization technique for compression, namely, exponential particle swarm optimization (ExPSO). In each iteration, ExPSO selectively identifies the best and fundamental weights of the model, mitigating potential damage resulting from compression. Our technique facilitates a graceful return to the best-performing weights when the iteration produces suboptimal weights. This approach preserves model accuracy and creates opportunities for improvement rather than merely stabilizing performance.

## The proposed methodology

In this paper, we build a novel methodology to protect neural models against adversarial textual attacks. This methodology consists of four methods: batch-cumulative exponential particle swarm optimization (BC-ExPSO) approach to reduce the risk of overfitting to specific batch characteristics; weight compression approach to avoid the time and memory consumption of the model; stochastic multi-expert (SME) approach to increase the resistance of the model to adversarial attacks; and multi-version compressed neural network training (MVC-NNT) approach to enhance the training efficiency and generalization capabilities of the detection method. Incorporating these approaches strengthens defenses against adversarial attacks, improves optimization, and maximizes resource utilization.

**Figure 2:**
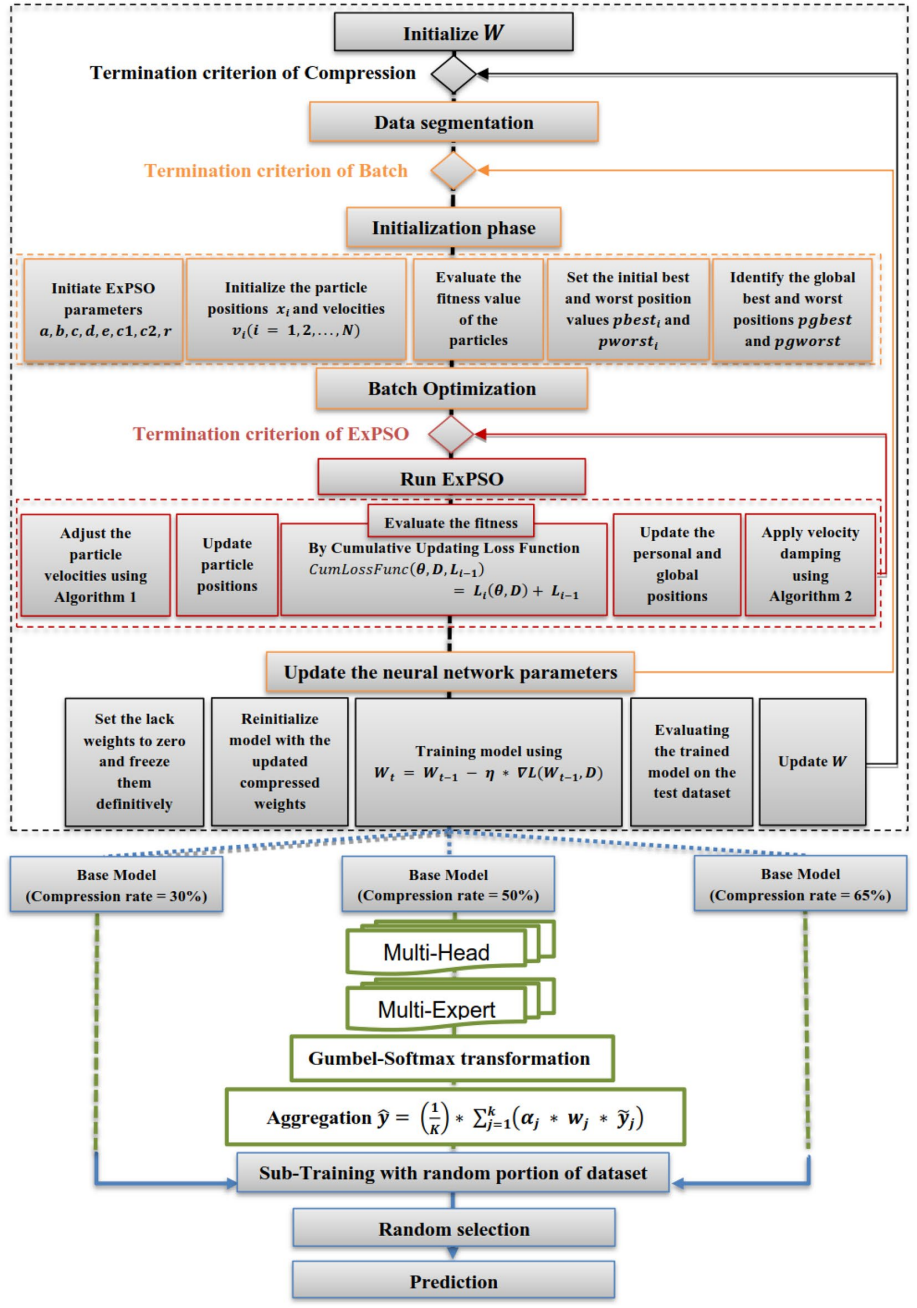
Flowchart of the proposed methodology.

The overall methodology is depicted in Fig. 2, beginning with the compression phase. During this phase, the weights of the GPT undergo optimization through a weight compression algorithm, illustrated by the black flows. This algorithm utilizes batch cumulative strategies (yellow flows) based on ExPSO (red flows), resulting in three distinct base models, each with varying compression percentage rates (30%, 50%, 65%). Following the compression phase, the multi-version training approach is implemented. It associates the three compressed model versions with a multi-expert system (green flows). Each model underwent training with different dataset partitions, yielding three trained models with distinct sets of weights and compression percentages but comparable performance. In the comprehensive model, one of the trained models is randomly selected for the final prediction (blue flows). The incorporation of stochastic methods introduces increased complexity, increasing perplexity levels and, consequently, reducing the likelihood of successfully inducing generalization changes, providing a defense mechanism against potential attackers.

### Batch-cumulative exponential Particle Swarm Optimization (BC-ExPSO)

In this section, a cumulative performance approach is used for optimizing the deep neural network. By considering the model's performance over multiple batches, the optimization method becomes more stable, and the risk of overfitting to specific batch characteristics is reduced. We initially explored the PSO^60^ optimizer, which exhibits powerful exploration capability within the solution space, leveraging swarm intelligence for rapid convergence. However, this approach may struggle with local exploitation^55^, heavily relying on the initial swarm configuration and requiring careful parameter tuning^54^. Additionally, its exploration might lead to prolonged computation times due to excessive exploration. On the other hand, gradient descent (GD) excels in exploiting local search for obtaining local optima; this method is deterministic, computationally efficient, and widely adopted in machine learning^48^. However, it may become stuck in local minima, become sensitive to the initial point, and need help with nondifferentiable or discontinuous objective functions^61^. To overcome these challenges, we use an advanced version of PSO^60^ called exponential particle swarm optimization for global optimization. Comparative analyses with various well-known heuristic search algorithms on 29 benchmark problems reveal that ExPSO significantly contributes to convergence velocity and optimization accuracy^62^. ExPSO employs an exponential search strategy, allowing particles to make leaps in the search space, effectively exploring the entire space and mitigating the limitation of GDs (trapped in local minima). The algorithm divides the swarm into three equal subpopulations $$(N1, N2$$ and $$N3)$$ for balanced exploration and exploitation and incorporates a dynamic cognitive parameter and inertia weight strategy for optimal convergence^63,64^. This division enhances convergence and allows for improved adaptation to various regions of the solution space.

**Figure 3:**
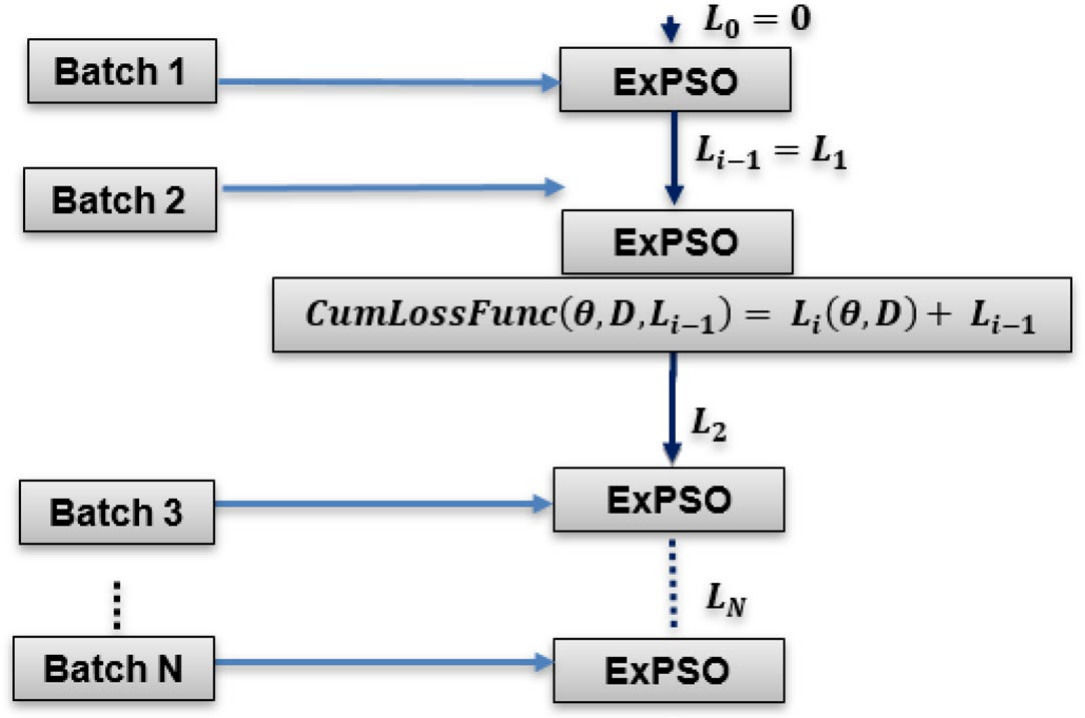
Batch-cumulative exponential Particle Swarm Optimization process.
  The batch loss ${L}_{i}\left(\theta , D\right)$ for multiclass classification is calculated using the binary cross-entropy loss formula, which can be summarized as follows:
  2 $$L\left(\theta , D\right)= - \frac{1}{N}\sum_{i=1}^{N}\sum_{j=1}^{C}yij\cdot log\left(f\left(\theta ,xij\right)\right),$$
  where $N$ is the number of samples in the batch, $C$ is the number of classes, *y*_ij_ represents the ground truth probability distribution for the *i*th sample, and the function *f*(*θ*, *x*_ij_) is the model's output, which represents the predicted probability distribution over all classes for the input *x*_i_.
  During each iteration of ExPSO, particles undergo comprehensive updates involving their positions, velocities, and best-found positions. This iterative process entails several key steps:
  The particle velocities are adjusted using Algorithm 1, which involves the integration of personal and global factors related to the best and worst positions ($pbest,gbest,pworst,gworst$), as well as the consideration of the inertia weight ($w$), a random vector (${R}_{vec}$), and predefined parameters ($a,b,c,d,e$) representing exponential weight, cognitive acceleration coefficients for the best and worst cases, and social acceleration coefficients for the best and worst cases.
  A constant k within the range of $(0, 1)$ is set to control the reduction in velocity. Subsequently, velocity constraints are enforced using ${v}_{i} =max \left({v}_{i}, {v}_{min}\right)$ and ${v}_{i} = min ({v}_{i}, {v}_{max})$, with a designated velocity range of $[{v}_{min}, {v}_{max}]$.
  The particle positions are updated by ${x}_{i} ={x}_{i}+ {v}_{i}$, ensuring that they remain within the bounds where ${x}_{i}=max ({x}_{i}, {x}_{min})$ and ${x}_{i}= min ({x}_{i}, {x}_{max})$. Here, ${x}_{min}$ and ${x}_{max}$ denote the lower and upper limits of the variables, respectively. Figure 4 shows an example in which each particle considers its previous velocity, global best position (social component), and personal best position (cognitive component) to determine the new velocity and update its position.

After dividing the data into batches, ExPSO optimizes the model parameters and iteratively updates the cumulative updating loss function. The next steps outline this method:

1. *Data segmentation:* The dataset is partitioned into more compact segments. The specific size of each segment is determined by the available resources, including memory and computational capabilities.
2. *Initialization phase:*
  The ExPSO process is initiated by configuring crucial parameters such as $a, b, c, d, e, {c}_{1}, {c}_{2},$ and $r$.
  Random and uniform initialization is introduced for the particle positions and velocities, denoted as ${x}_{i}$ and ${v}_{i} (i = 1, 2, . . . , N),$, respectively.
  The fitness values of the particles are evaluated based on their positions and initial best and worst position values, denoted as ${pbest}_{i}$ and ${pworst}_{i}$, respectively.
  The global best and worst positions across the entire particle swarm are identified and denoted as $pgbest$ and $pgworst,$ respectively.
3. *Batch optimization*: As shown in Fig. 3, the ExPSO algorithm^62^ is used for each batch to optimize the model parameters (positions) while keeping track of the best-found position. A cumulative updating loss function $CumLossFunc$ is employed to evaluate the model's performance with a given set of parameters. This function considers the current model parameters $\theta$ (particle position), the training data batch $D$, and the previous loss value from the previous iteration ${L}_{i-1}$. The algorithm updates the batch loss by adding the current loss ${L}_{i}\left(\theta , D\right)$ to the previous loss ${L}_{i-1}$, allowing the ExPSO algorithm to consider the cumulative performance of the model during optimization. The cumulative updating loss function is expressed in Eq. (1).
  1 $$CumLossFunc\left(\theta , D, {L}_{i-1}\right)= {L}_{i}\left(\theta , D\right)+ {L}_{i-1}$$

**Figure 4:**
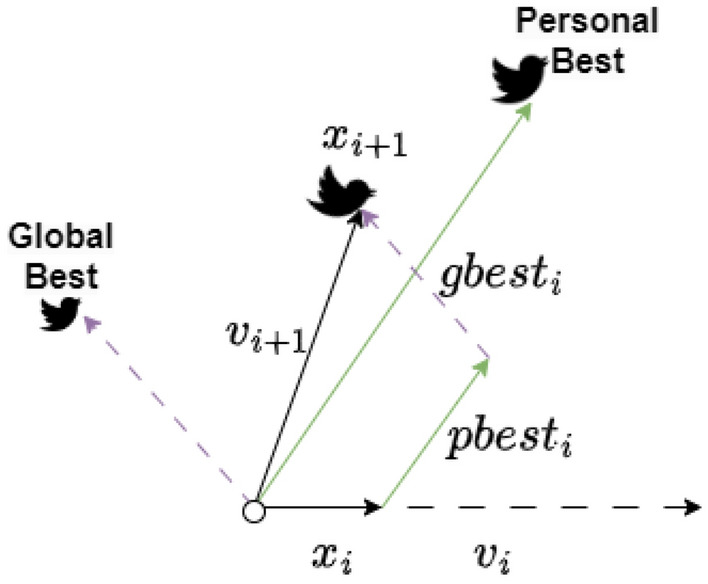
The position update process in ExPSO.

  Next, the fitness value is evaluated, and the personal and global positions for the best/worst cases are updated.
  The dynamic parameter $a$ is adapted by using $a =r \cdot  a$, where $r$ represents the damping coefficient, which belongs to the range $r \in [0.5, 2]$.
  Finally, the application of velocity damping, as described in Algorithm 2, is used to conclude the iterative process.
4. *Parameter Updates for Deep Neural Network Optimization*: The Deep Neural Network parameters are updated by the optimal set of parameters obtained from the global best position in step 3. The optimization process is iterated for multiple batches to further improve the model's performance. This approach promotes the generalization of unseen data, as the model learns to perform well across various data distributions rather than being biased toward any single batch. Additionally, optimizing overall cumulative performance enables faster convergence and improves model performance in fewer iterations than training each batch independently, which is valuable when computational resources are limited. Furthermore, by balancing learning dynamics across batches, the model is prevented from getting stuck in local optima^65^ associated with individual batches.

**Algorithm 1:**
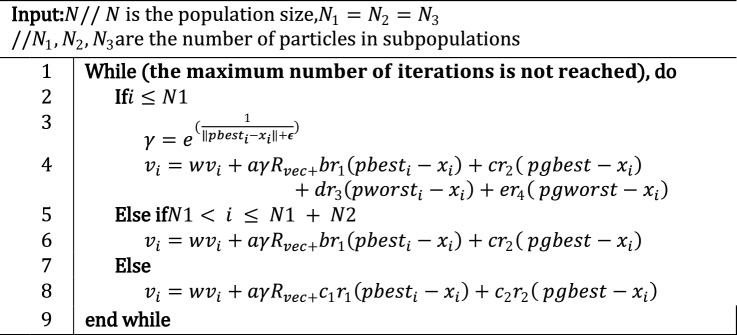
ExPSO Subpopulations Algorithm.

**Algorithm 2:**
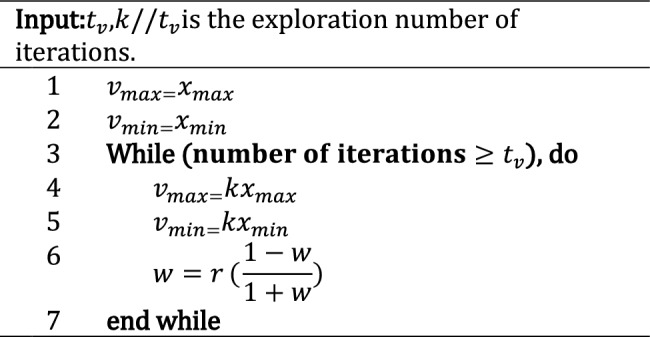
Velocity Controller Algorithm.

### Weights compression of the deep neural network

In this section, a novel algorithm (see Algorithm 3) is designed to optimize the weights of the deep neural network while considering compression. The algorithm aims to find a balance between accuracy and model size by iteratively compressing certain percentages of positive and negative weights while optimizing the model's accuracy. Initially, the model's weights $W$ are initialized. Next, the algorithm applies the BC-ExPSO method for each compression to optimize the model weights. Then, the algorithm identifies positive and negative weights separately, sorts them, and selects the first $P1$ and $P2$ percent of positive and negative weights, respectively. These selected weights are set to zero and frozen definitively to perform the compression. The DNN is then reinitialized and retrained with the previous compressed weights ${W}_{t-1}$. During the training process, the model's parameters ${W}_{t}$ (the current compressed weights) are updated based on the gradients computed during backpropagation using the previously compressed weights, as shown in Eq. (3).

3 $${W}_{t} = {W}_{t-1} - \eta * \nabla L\left({W}_{t-1}, D\right),$$

where $\nabla L({W}_{t-1}, D)$ is the gradient of the loss function for the compressed weights at compression $t-1$ and $\eta$ is the learning rate. Next, the algorithm assesses the performance of the compressed deep neural network on the test dataset. Finally, the variable $W$ is updated if the new accuracy is superior to or equal to the previous values.

**Figure 5:**
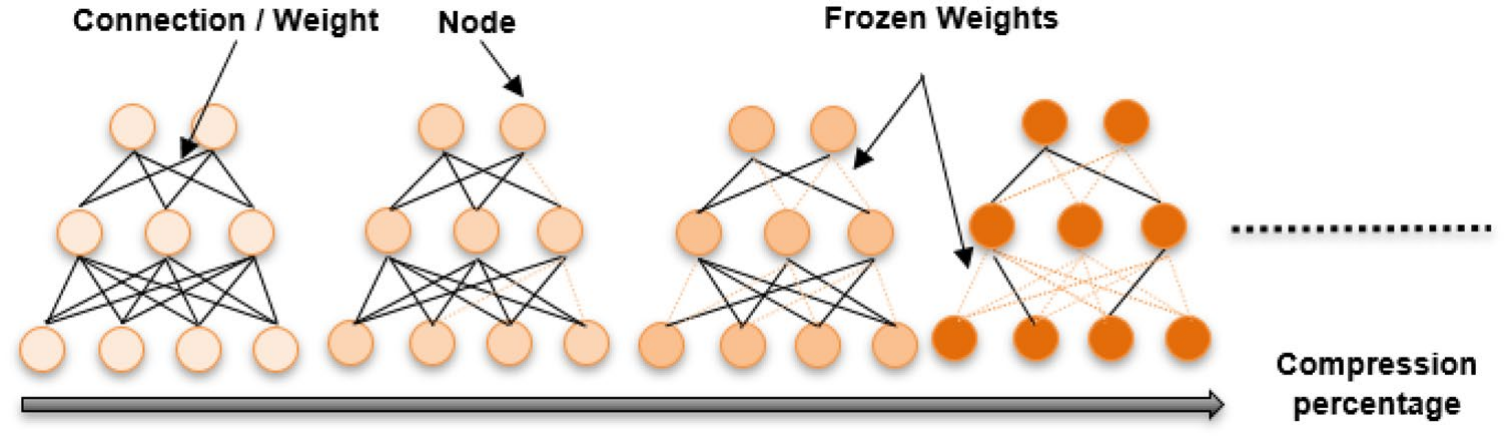
The frozen weight technique across various compression percentages.

**Figure 6:**
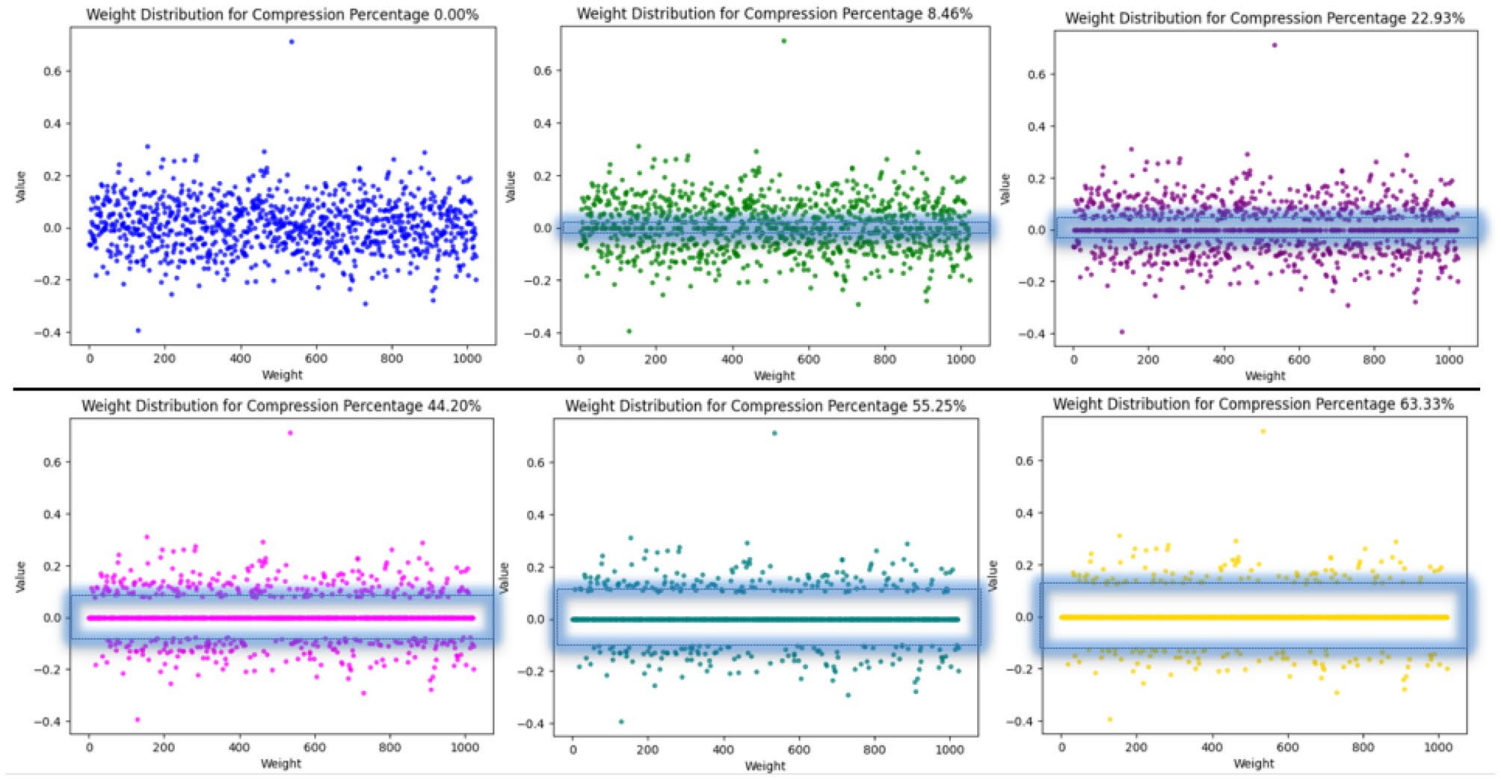
Weight distributions across various compression percentages.

The deep neural network weight is a crucial parameter that defines the strength of connections between nodes. When the compression algorithm sets the weight of a connection to zero, it essentially eliminates the impact of the connection on the output of the target node. This action disconnects the influence of that particular connection. However, the nodes remain within the network and can potentially contribute to computations through their connections to other nodes. Figure 5 exemplifies the freezing weight technique across different compression iterations. In tandem, Fig. 6 offers insights into the distribution of weights across various compression percentages through the proposed approach. The initial weights of the generative pre-trained model before any compression are depicted in the first figure. As compression initiates, the noticeable increase in frozen weights, drawn from both positive and negative initial values within the range of [− 1, 1], represents the lower and upper boundaries of the ExPSO algorithm. This process simplifies decision boundaries, contributing to improved generalizability. Less convoluted decision boundaries are more likely to capture underlying patterns in the data rather than fitting to noise or specificities of the training set, enhancing the model's performance on new, unseen data^40^.

**Algorithm 3:**
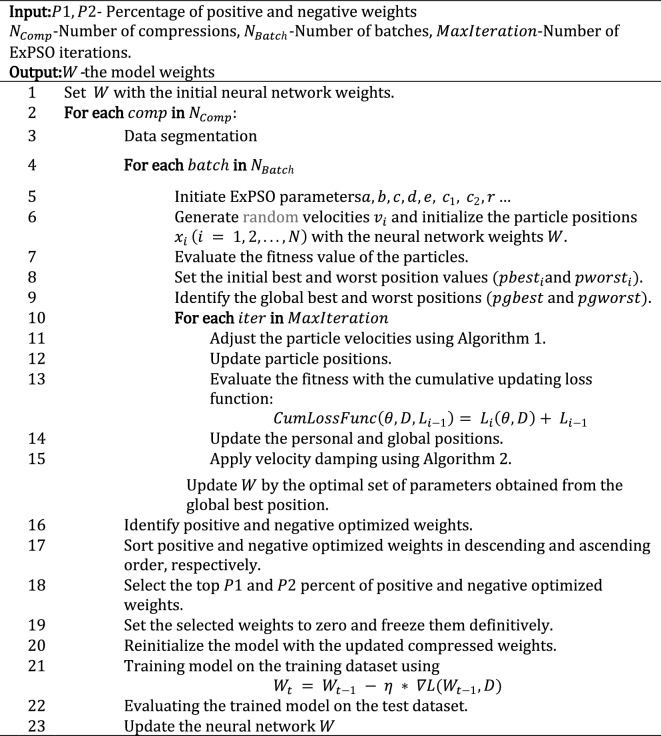
Weights Compression of Deep Neural Network algorithm.

Setting some weights to zero could speed up inference by reducing the multiplications and additions required during the forward pass. To illustrate this concept, let us consider a fully connected layer within a deep model containing a single neuron. This neuron has $n$ input connections, each represented by weights ${w}_{i}$, a bias term $b$, and $y$ as the output of the neuron. The computation for this neuron can be expressed in Eq. (4).

4 $$ y = \sum\limits_{{i = 1}}^{n} {w_{i} x_{i}  + b}  $$

However, when some weights are intentionally set to zero (for example, $k$ weights), the computation simplifies to Eq. (5).

5 $$ y = \sum\limits_{{i = 1}}^{{n - k}} {w_{i} x_{i}  + b}  $$

In this scenario, we effectively skip the multiplications by zero, reducing the number of operations. The degree of speedup in inference time depends on the ratio $k/n$. For instance, if half of the weights in the layer are set to zero $\left(k=\frac{n}{2}\right)$, we can reduce the number of multiplications in that layer by half, potentially resulting in a noteworthy acceleration during inference.

### Stochastic Multi-expert approach (SME)

A novel stochastic multi-expert approach is built to protect against adversarial attacks; this approach focuses on enhancing GPT, denoted as $f(x,\theta )$, by exclusively modifying its final layer. The result is the model’s conversion into an ensemble of several expert predictors, each associated with stochastic weights. Depending on the input, these predictors are designed to be strategically selected with different weights during inference. First, we transform $f(\cdot )$ into a randomized ensemble of different heads. To facilitate a stochastic weighted ensemble among heads, we extend the last layer of $f(\cdot )$, which is typically a fully connected layer, to an ensemble of $K$ prediction heads, denoted as $H={\{h(\cdot )\}}_{j}^{k}$. Each head, denoted as $hj\left(\cdot \right)$ and parameterized by ${\theta }_{{h}_{j}}$, serves as an expert predictor. It receives a feature representation learned by $f\left(\cdot \right)$ up to the second-last layer and produces a prediction logit score, as depicted in Eq. (6).

6 $${h}_{j} : f\left(x,{ \theta }_{L-1}^{*}\right)\in {R}^{Q}\to {\widetilde{y}}_{j} \in {R}^{M },$$

where ${\theta }_{L-1}^{*}$ are fixed parameters of $f$ up to the last prediction head layer, $L$ is the number of head layers, $Q$ is the size of the feature representation $x$ generated by the base model$,$ and $M$ is the number of labels.

**Figure 7:**
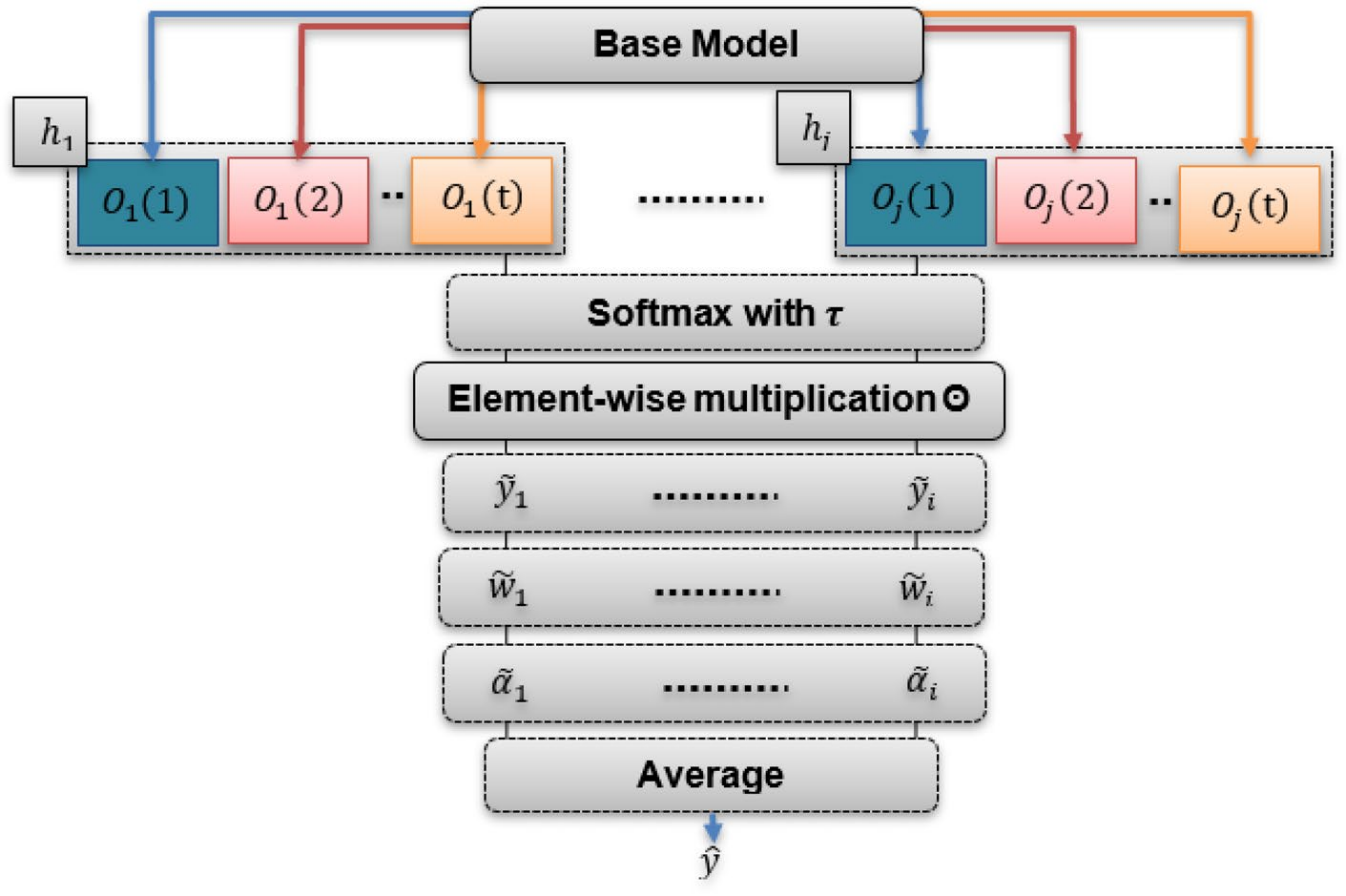
Stochastic multi-expert process.

Our primary focus is optimizing the diversity inherent in how each head generates predictions. To address this objective, the average expert architectures of each head were considered. This averaging mechanism allows the model to incorporate knowledge from various architectures, capturing different aspects of the data and increasing its generalization capabilities. Figure 7 illustrates the structure of this approach, which employs the Gumbel-Softmax transformation method, which involves sampling from a categorical distribution using Gumbel-distributed noise. The Gumbel noise ${G}_{j}$ is created by utilizing ${U}_{j}$, which represents samples from the uniform distribution within the range of (0, 1). The noise is derived from a Gumbel distribution and generated by applying the function $-log\left(-log\left({U}_{j}\right)\right)$. Then, the softmax function is applied to the ${O}_{j}$ vector with a temperature parameter $\tau$, as illustrated in Eq. (7).

7 $$softmax\left({O}_{j} ,\tau \right)=\left[\frac{{e}^{{O}_{j}\left(1\right)/\tau }}{\sum_{t=1}^{T}{e}^{{O}_{j}\left({\text{t}}\right)/\tau }},\frac{{e}^{{O}_{j}\left(2\right)/\tau }}{\sum_{t=1}^{T}{e}^{{O}_{j}\left({\text{t}}\right)/\tau }},\dots,\frac{{e}^{{O}_{j}\left({\text{T}}\right)/\tau }}{\sum_{t=1}^{T}{e}^{{O}_{j}\left({\text{t}}\right)/\tau }}\right],$$

let ${O}_{j}$ represent a vector of values $\{{O}_{j} (1), {O}_{j} (2),\dots , {O}_{j} (T)\}$ for the ${j}^{th}$ possible architecture to be selected for ${h}_{j} \in H$.

After the softmax probabilities are obtained, as depicted in Eq. (8), they are combined with the Gumbel noise ${G}_{j}$ to introduce stochasticity and enable exploration during selection. Here, ⊙ represents elementwise multiplication.

8 $${\widetilde{y}}_{j}=softmax({O}_{j},\tau )\odot {G}_{j}$$

Finally, the method outlined in Ref.^12^ is adopted, where randomness is introduced in the process by assigning prediction heads with stochastic weights in both the training and inference phases. More precisely, we employ the aggregation mechanism, as illustrated in Eq. (9).

9 $$\widehat{y}= \left(\frac{1}{K}\right)* \sum_{j=1}^{k}\left({\alpha }_{j} * {w}_{j} * {\widetilde{y}}_{j}\right),$$

where the weight ${w}_{j}$ scale ${\widetilde{y}}_{j}$ corresponds to the expertise of the head $j$ for the current input $x$. The scalar ${\alpha }_{j}$, ranging between 0 and 1, probabilistically determines how much the weight ${w}_{j}$ contributes. Equations (10) and (11) are employed to compute the values of $w$ and $\alpha .$ These vectors belong to the vector space ${R}^{K}$ contain the scalars ${w}_{j}$​ and ${\alpha }_{j}$, ​respectively. Additionally, $\widetilde{y} \in {R}^{\left(K\times M\right)}$ represents the concatenation of ${\widetilde{y}}_{j}$​ vectors returned from each head, while $W \in {R}^{(K\times M+Q)\times K}$ and $b \in {R}^{K}$ serve as the trainable parameters.

10 $$w = {W}^{T} (\widetilde{y} \oplus f(x, { \theta }_{L-1}^{*})) + b$$

11 $$\alpha = softmax((w + G)/\tau )$$

### Multi-version compressed neural network training (MVC-NNT)

**Figure 8:**
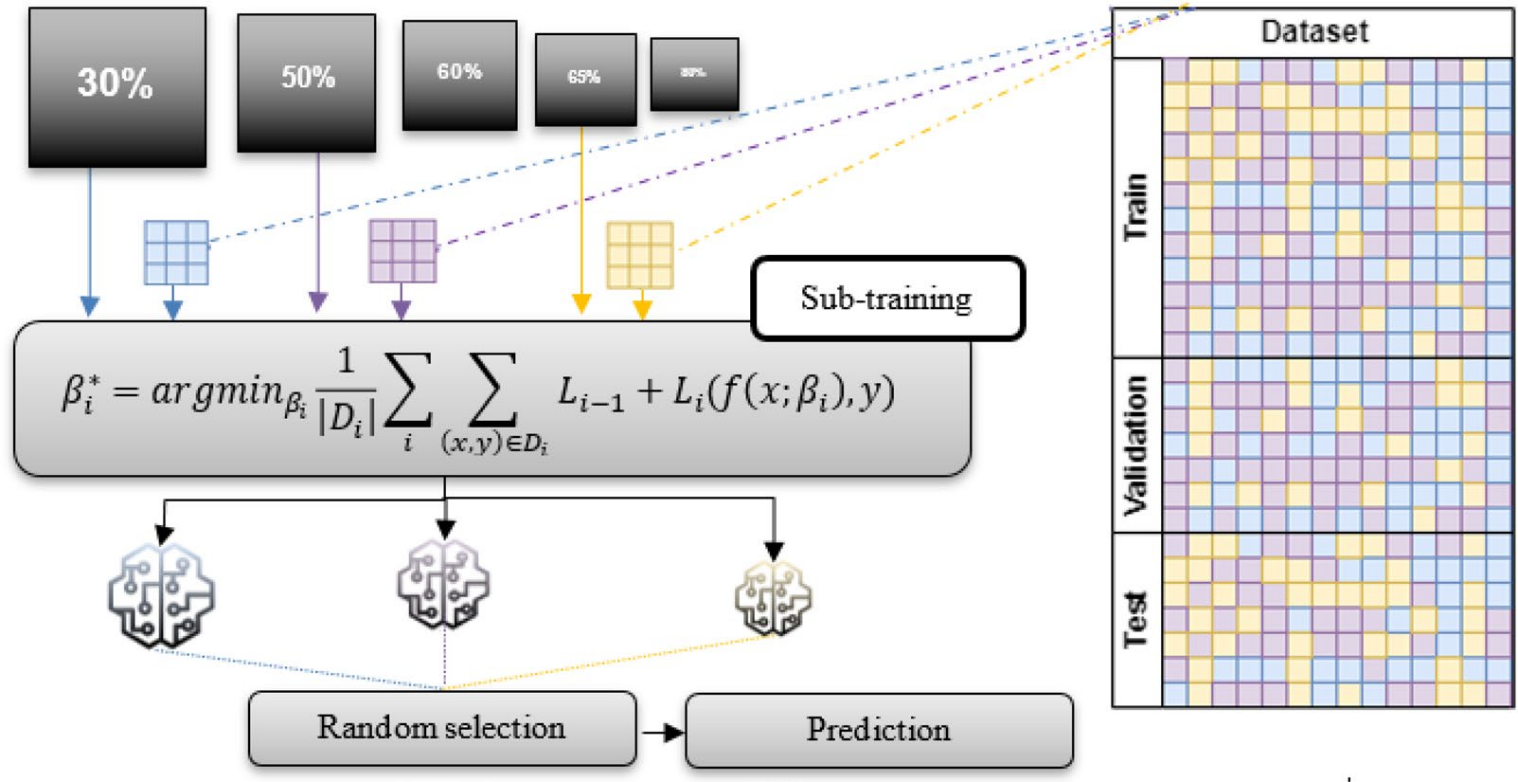
Multi-version compressed neural network training for the enhanced adversarial attack detection process.

To improve the training efficiency and enhance the generalization capabilities of our method for detecting adversarial attacks, we adopted an approach that employs multiple versions of the base model, each utilizing distinct compression values. The multi-version training process comprises training three variations of the base model on distinct dataset partitions, each with different compression rates, as depicted in Fig. 8. First, the dataset is partitioned into three subsets: ${D}_{1},{D}_{2},{and D}_{3}$. Then, a common NN architecture $f$ is defined to be employed across all base model versions. Subsequently, the final predictions are made through a random selection method among the base model versions (${\beta }_{1}^{*}$, ${\beta }_{2}^{*},$ and ${\beta }_{3}^{*})$. For each version $i$, the training objective is to minimize the loss of function $L$ for the Neural Network parameters in Eq. (12):

12 $${\beta }_{i}^{*}={argmin}_{{\beta }_{i}}\frac{1}{\left|{D}_{i}\right|}\sum_{(x,y)\epsilon {D}_{i}}L(f\left(x;{\beta }_{i}\right),y),$$

${{\text{here}},\beta }_{i}^{*}$ represents the optimal set of parameters for the base model version $i$, which are the values of ${\beta }_{i}$ that minimize the average loss over the dataset ${D}_{i}$. The variables ${\beta }_{i}$ are the updated compressed base model parameters. The values $x$ and $y$ represent the input data and the target labels, respectively.

## Experimental evaluation

This section provides a detailed account of the datasets utilized, the metrics applied, and the experimental settings configured to evaluate our proposed methodology. We also discussed the results of these evaluations and outlined the limitations and potential avenues for future research.

### Datasets

Our research paper aims to evaluate the efficacy of our defense methodology against adversarial attacks. For that purpose, we utilized three publicly available datasets: Clickbait detection (CB)^66^, Hate Speech Detection (HS)^67^, and Movie Reviews classification (MR) datasets^68^. Table 4 provides an overview of the statistical information about these experimental datasets.

**Table 4:** Overview of the experimental datasets and partitioning information.

Dataset	Class	Vocabulary	Examples
MR	Binary	19,000	11,000
CB	Binary	25,000	32,000
HS	Multiclass	35,000	25,000

**Figure 9:**
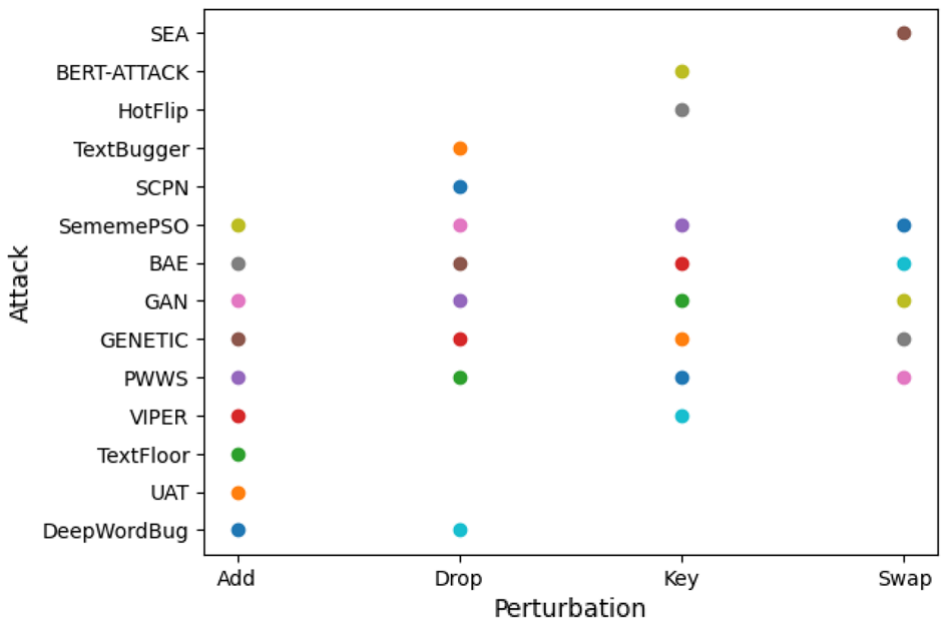
Word perturbation methods for 14 adversarial attacks.

**Figure 10:**
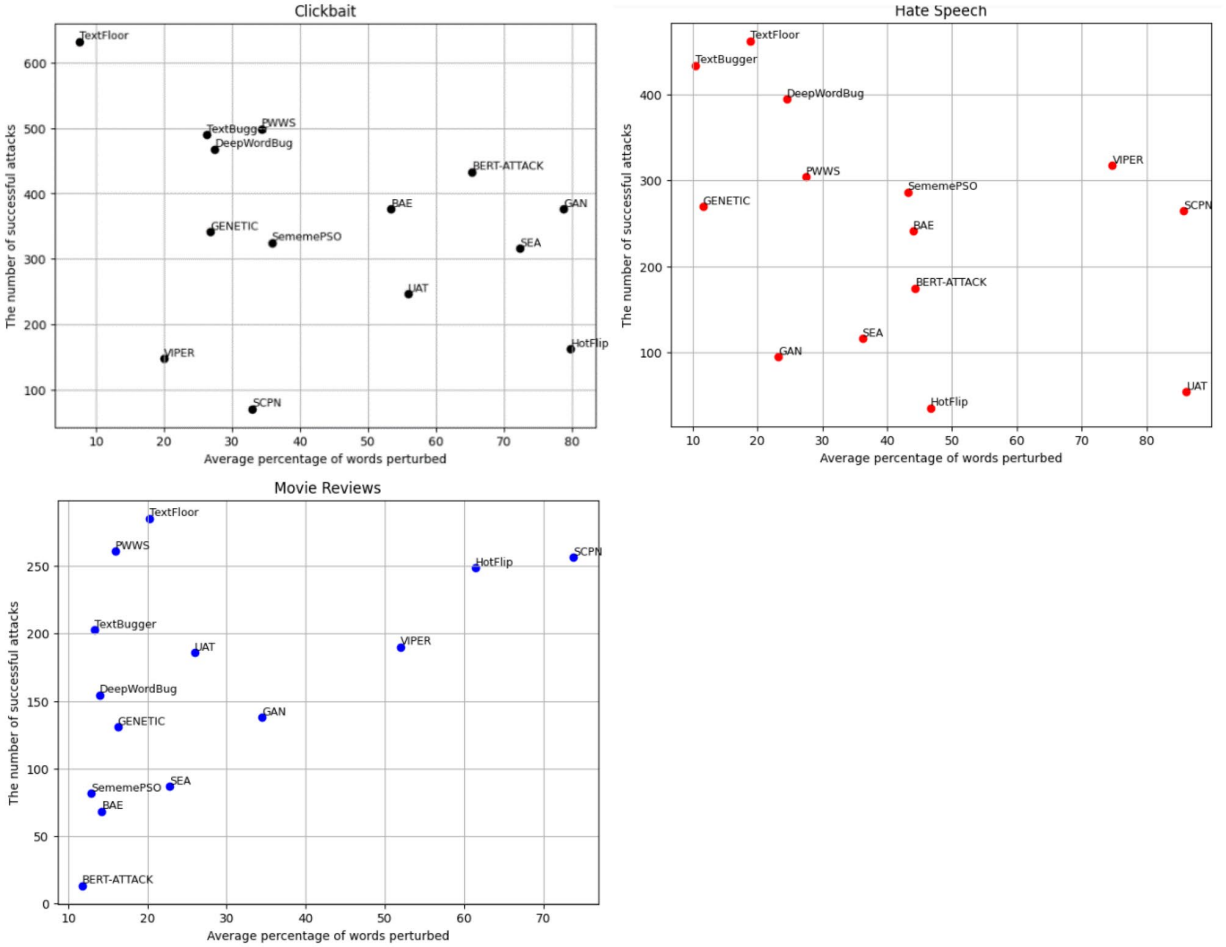
The average percentage of words perturbed per successful attack: a visual representation of the application of 14 attacks, considering their success rates and percentages of perturbation on three datasets using a simple pre-trained GPT.

The datasets above undergo adversarial attacks to generate diverse, challenging samples or perturbations. To achieve this goal, we employed the OpenAttack framework^22^, a Python-based open-source toolkit specifically designed for conducting textual adversarial attacks. To increase the complexity of our datasets, we meticulously select the applied adversarial attacks through a comprehensive review of various attacks based on multiple criteria. Figure 9 visually categorizes attacks based on their perturbation methods. For instance, the SEA attack relies solely on swapping perturbations, whereas the DeepWordBug attack utilizes addition and dropping perturbations. In Fig. 10, we provide a visual representation of the application of 14 attacks, considering their success rates and percentages of perturbation on three datasets using a simple pre-trained GPT. Notably, TextFloor, TextBugger, DeepWordBug, and PWWS from OpenAttack perturb only 5–40% of words in the input but yield more successful attacks than the other methods in the three datasets. Following this analysis, we utilized 14 attacks employing different perturbation methods, with variations in perturbation percentages and diverse success rates.

### Metrics

We aim to provide a thorough assessment of our methodology. To accomplish this, we employ widely recognized metrics commonly used in the literature.

To assess the prediction performance, we utilized the weighted F1-score and accuracy, as defined in Eqs. (13) and (14), respectively. These metrics evaluate the model's accuracy across diverse classes. The prediction accuracy under adversarial attacks is presented to gauge the robustness of our methodology (the ratio of failed attacks to the total number of examples^69^). A failed attack is recorded only when the adversary's attempt to perturb an input fails, resulting in the label remaining unchanged for correctly predicted clean examples.

13 $$ F1{\text{-}}score = 2\frac{{(precision*recall)}}{{precision + recall}} $$

14 $$Accuracy=\frac{True\;Positive+True\;Negative}{FalsevPositive+False\;Negative+True\;Positive+True\;Negative}$$

To evaluate the effectiveness of the optimization method, we conduct a dynamic loss test in which we utilize categorical cross-entropy, a suitable loss function for multiclass classification tasks, as defined in Eq. (15). Next, we conduct two nonparametric statistical tests. Initially, we employ the Friedman test to comprehensively compare the performance of each algorithm across a set of functions^70^. The procedure entails collectively ranking each row (or block) and examining the rank values by columns to gain insights into the algorithms' overall performance. Instead of using a set of functions in our experiment, we use the Friedman mean of the benchmarks of 23 functions, which contain unimodal, multimodal, and composite problems^55,56,62^. Second, we employ the Wilcoxon signed-rank test with a 5% significance level to assess the validity of the results, discern which algorithms perform better or worse than ExPSO, and gauge the meaningfulness of the improvements achieved by ExPSO.

15 $$Loss=-\sum_{i=1}^{n}{y}_{i}\cdot {\text{log}}\widehat{{y}_{i},}$$

where $n$ is the number of data points, ${y}_{i}$ is the actual value, and $\widehat{{y}_{i}}$ is the predicted value.

To gauge the effectiveness of the compression technique, we assess the model's performance using processing speed, which is measured through two key performance indicators, namely, Throughput and Inferences Per Second (IPS), as illustrated in Eqs. (16) and (17), respectively, where Throughput signifies the rate at which a text classification model processes and classifies a batch of textual documents^71^. Moreover, the IPS quantifies the number of inferences, such as predictions, that the model can generate per second.

16 $$Throughput=\frac{Number\;of\;units\;produced}{Time\;period}$$

17 $$IPS=\frac{Number\;of\;Inferences}{Time\;taken\;for\;the\;inferences}$$

For a more comprehensive evaluation, we introduce another metric known as perplexity. This metric is crucial for assessing language models such as the GPT, as it evaluates the model's accuracy in predicting sequences of tokens^72^. Perplexity entails encoding the entire test dataset using the model's tokenizer, segmenting it into numerous token segments, and calculating the average language modeling loss. The resulting exponentiated number represents the reported perplexity, as illustrated in Eq. (18).

18 $$PPL=exp\left(\frac{1}{N}\sum_{i=1}^{N}-{\text{log}}P\left({w}_{i}\right)\right),$$

where $N$ is the sample's number of words (or tokens). $P\left({w}_{i}\right)$ is the probability assigned to the *i*th word by the model.

Additionally, we incorporate GPU and CPU speedup and latency metrics through Eqs. (19)–(21) to further assess the overall performance and efficiency of the compression technique. These metrics provide valuable insights into how the compression method impacts the speed and responsiveness of the model on different hardware platforms.

19 $$Speed Up=\frac{{Execution Time}_{Baseline}}{{Execution Time}_{Improved}}$$

20 $${Latency }_{GPU}=\frac{1}{{{\text{Throughput}}}_{GPU}}$$

21 $${Latency}_{CPU}={Time}_{CPU}+{Queueing}_{CPU},$$

where ${Queueing}_{CPU}$ is the time spent in the CPU queue before the task is executed and ${Time}_{CPU}$ is the time the CPU takes to execute the task.

### Experimental setting

Regarding hardware, CPU experiments were conducted on a standard Windows (v8.1) server with an Intel Core i7 processor. GPU experiments were performed using an online Intel framework with an Intel Data Center GPU Flex 140, employing a batch size of 8 sentences and a maximum sequence length of 512 tokens. All the software implementations were coded in Python (v3.7) using PyTorch (v1.5.1), NumPy (v1.19.1), and Scikit-learn (v0.21.3). The choice of the pre-trained model was GPT-2, primarily due to its readily available codebase. The selected GPT-2 model consists of 24 layers, a hidden size of 1024, 16 attention heads, and 345 million parameters^73^.

We have devoted careful attention to specifying hyperparameters at every stage of developing and assessing our proposal. These hyperparameters have been carefully selected to ensure optimal performance and generalization across various tasks. Table 5 lists the hyperparameters utilized in our approach and their corresponding values. In global optimization, the coefficients for both cognitive and social aspects are set to 2, emphasizing the influence of personal and social experiences on particle movement. The choice of − 1 for the worst-case coefficients introduces a competitive element among particles, promoting diverse exploration and contributing to efficient convergence. This configuration has been observed to facilitate effective convergence, particularly in complex and multimodal real-world optimization engineering scenarios^62,74^. The remaining values of the hyperparameters were chosen based on Refs. ^55^, in which the authors tested each parameter with different values to find the best value, ensuring the robust performance of ExPSO across a range of optimization scenarios. To determine the compression hyperparameters, we thoroughly reviewed the standard settings reported in the literature^17–19,57^. Drawing upon established practices, we systematically identified and chose the parameters that consistently produced optimal results across different experiments. On the other hand, when adopting optimization methodologies in the literature, such as PPSO^53^, FMPSO^54^, MSPSO^55^, and XPSO^56^, we use the same hyperparameters that have demonstrated superior performance. This approach allows us to ensure the stability and effectiveness of the optimization method by preserving configurations known to be robust and high-performing.

**Table 5:** Hyperparameter configurations for training and testing the overall methodology.

Processes	Hyperparameters	Parameters setting
ExPSO	Exponential weight parameter	$a = 2$
	Cognitive acceleration best-coefficient	$b = 2$
	Social acceleration best-coefficient	$c = 2$
	Cognitive acceleration worst-coefficient	$d = -1$
	Social acceleration worst-coefficient	$e = -1$
	Cognitive scaling parameter	${c}_{1} = -1$
	Social scaling parameter	${c}_{2} = 2$
	Inertia weight	$W = 0.9$
	Damping coefficient	$r = 0.9$
	Exploration number of iterations	$t = 10$
	Decreasing velocity coefficient	$k = 0.2$
	Number of particles of subpopulation 1,2, and 3	${N}_{1},{N}_{2},{N}_{3}=10$
Compression	Learning rate (We maintain a fixed learning rate for our initial evaluation. However, for optimization problems with complex landscapes, we recommend starting with a fixed learning rate and subsequently considering updates to it as needed)	$Lr= 3e-5$
	Epsilon	$Eps= 1e-8$
	Compression dimensionally	$D=50257*1024$
	Max iteration limit	$MaxIt = 100$
	Early stopping patience	$20\, epochs$
	Epochs	$100\, epochs$
	Lower bounds	$Lb = -1$
	Upper bounds	$Ub=1$
Multi-expert	Number of heads	$H=5$
	Number of experts	$L=3$
XPSO	$n =0.2, {Stag}_{max}=5, p =0.5$	
MSPSO	$X = 0.7298, {c}_{1} = {c}_{2} = 1.49445, {R}_{2} = 10$	
FMPSO	${c}_{H}=1.2, {c}_{m}= 0.7, {c}_{l} = 0.2$	
PPSO	Inertia weight	$w = 0$

### Results

For our multi-version compressed neural network training, the primary objective is to promote diversity among machine learning model parameters. This diversity is crucial for discouraging attackers from predicting model parameters effectively. The key strategy involves predefining a base model, such as GPT (Base), and compressing it to create three distinct models (Models 1, 2, and 3) with compression percentages set at 30%, 50%, and 65%, respectively.

**Figure 11:**
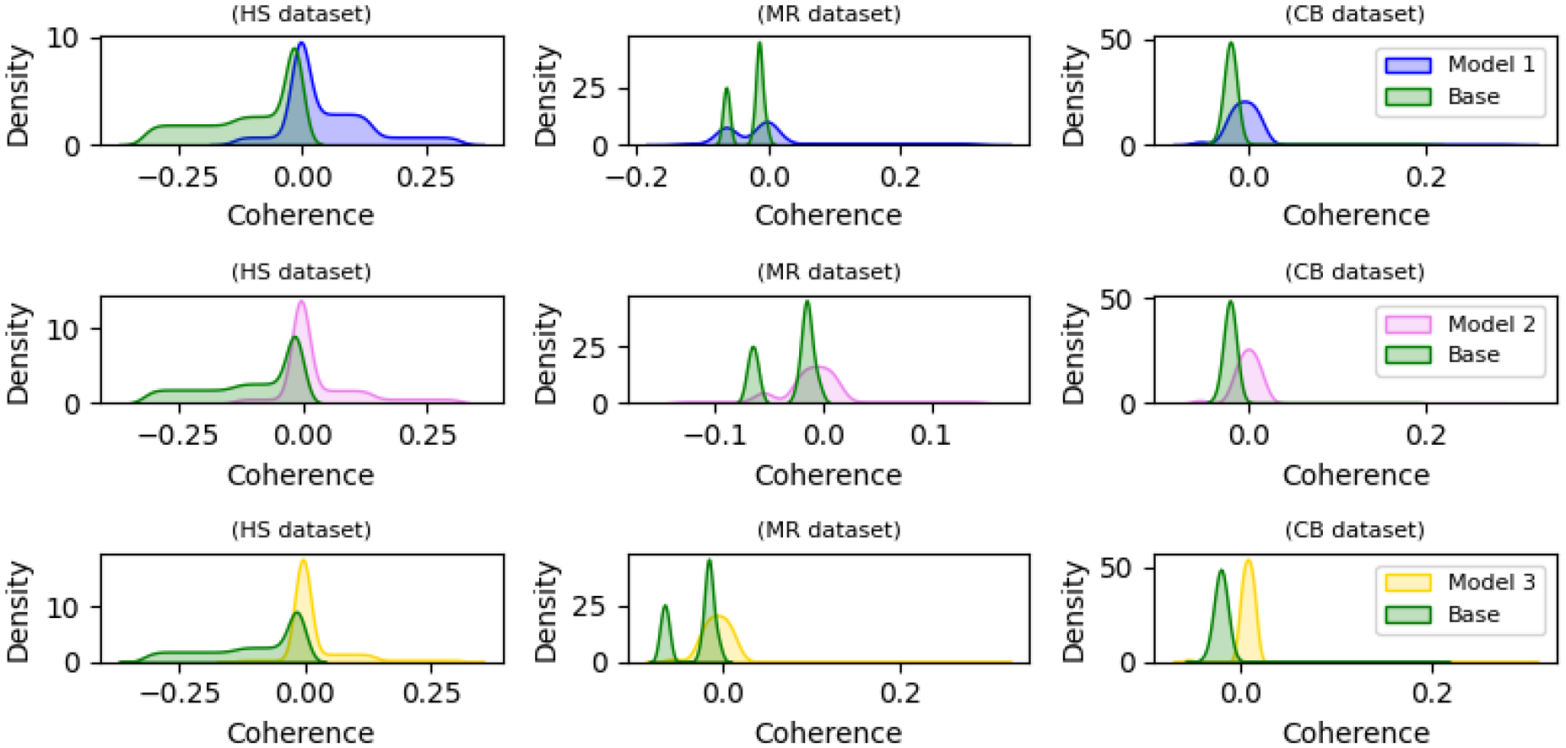
Comparative coherence analysis of the base model and compressed models (Models 1, 2, and 3) on the HS, MR, and CB datasets.

The focus is on preventing excessive similarity, especially in gradient vectors, among the models within an ensemble during training. Figure 11 evaluates the alignment degree among gradient vectors in various datasets. The evaluation involved comparing the distribution of coherence values for various target models, including the base model (Base) and the compressed models (Models 1, 2, and 3). Figure 11 illustrates those combinations of Base + Model 1, Base + Model 2, and Base + Model 3 exhibit lower coherence, particularly in the case of Base + Model 3. This observation implies that the compressed models demonstrate more misaligned gradient vectors, indicating reduced correlation among their gradients with the base model in different datasets. This demonstrates that the proposed multi-version compressed neural network training method effectively creates ensembles with diverse models and misaligned gradients, enhancing adversarial robustness.

In our evaluation, we assess the predictive performance of our methodology in the absence of adversarial attacks. As depicted in Table 6, our methodology consistently maintains superior F1 scores (0.94) on average across all the datasets. Notably, even when compared with the SHIELD, our methodology achieves a marginal performance reduction in the context of the Hate Speech dataset. However, this decrease is practically insignificant when contrasted with our methodology's substantial gains in adversarial robustness (expounded upon below). Importantly, our methodology showed a notable 1.8% enhancement in the F1 score across all the datasets compared to SHIELD model results.

**Table 6:** Comparison of model performance on various datasets (MR, HS, and CB) with baseline methods such as DT, ADP, Mixup, AdvT, ScRNN, and SHIELD.

Model/dataset	MR	HS	CB	AVG
+ DT	0.89	0.86	0.93	0.91
+ ADP	0.87	0.88	0.96	0.90
+ Mixup	0.78	0.87	0.97	0.87
+ AdvT	0.77	0.89	0.92	0.88
+ ScRNN	0.80	0.86	0.95	0.87
+ SHIELD	0.89	**0.94**	0.97	0.93
+ Our	**0.90**	0.93	**0.98**	**0.94**

Significant values are in bold and underline.

Table 7 summarizes the performance of our methodology under various adversarial attacks across different datasets. The methodology consistently exhibited enhanced performance after these attacks, with an average improvement of 17%. Attacks such as SCPN and SEA achieve perfect scores, indicating high effectiveness, while others such as BERT-ATTACK and DeepWordBug yield mixed results. The methodology exhibited the strongest performance on the Movie Reviews dataset, demonstrating a remarkable 25% relative improvement. This dataset is closely followed by the Hate Speech dataset, with a substantial 17% enhancement, followed by the Clickbait dataset, which achieves a commendable 9% boost. For the VIPER and UAT attacks, the employed approach neither resulted in improvement nor a significant performance drop. Intuitively, one might assume that word-level models would exhibit greater robustness, as they can leverage the surrounding context. Surprisingly, we observe that these attacks are more susceptible to attacks. To understand why, consider the word 'beautiful', which can be altered in limited ways for word-level models, resulting in either a UNK token or an existing vocabulary word. In contrast, character-level models treat each unique character combination differently. This diversity provides attackers with more potential variations to exploit. Consequently, this justifies the observed decrease in accuracy for certain character-level attacks (VIPER and UAT).

**Table 7:** Results of the adversarial attack mitigation technique (before and after) on multiple datasets.

Attack type	Dataset	Movie reviews		Hate speech		Clickbait	
	Attack	Before	After	Before	After	Before	After
Word-level	TextFloor	0.27	**0.79**	0.32	**0.68**	0.65	**0.85**
	PWWS	0.21	**0.58**	0.35	**0.60**	0.66	**0.80**
	GENETIC	0.15	**0.63**	0.23	**0.58**	0.58	**0.76**
	SememePSO	0.40	**0.76**	0.67	**0.79**	0.79	**0.90**
	BAE	0.85	**0.99**	0.94	**0.96**	**0.98**	0.97
	BERT-ATTACK	0.8	**0.71**	0.24	**0.75**	0.52	**0.78**
	HotFlip	0.47	**0.84**	0.71	**0.88**	0.81	**0.85**
Sentence-level	SEA	**1**	**1**	**1**	**1**	**1**	**1**
	GAN	0.63	**0.89**	0.60	**0.66**	0.54	**0.56**
	SCPN	**1**	**1**	**1**	**1**	**1**	**1**
Char-level	DeepWordBug	0.58	**0.92**	0.74	**0.83**	0.62	**0.85**
	VIPER	**0.94**	0.93	**0.99**	0.97	**0.98**	0.97
	UAT	0.11	**0.74**	0.18	**0.35**	**0.39**	0.36
	TextBugger	0.5	**0.64**	0.25	**0.65**	0.61	**0.74**
Average		0.565	**0.815**	0.587	**0.764**	0.723	**0.813**
Relative $\downarrow$↑%		↑25%		↑17%		↑9%	

Significant values are in bold.

Table 8 illustrates the impressive effectiveness of our methodology across multiple datasets under the TextFooler, TextBurger, DeepWordBug, and PWWS attacks. These attacks are selected because they are among the strongest attacks and provide foundation mechanisms upon which other attacks are built (refer to Fig. 10). Notably, our methodology consistently outperforms the baseline approach, resulting in substantial performance improvements in various datasets. This finding suggested that our methodology has the potential to significantly enhance model resilience to adversarial attacks, with an average improvement of 13–49% compared to the baseline.

**Table 8:** Performance comparison of various defense techniques against four adversarial attacks. The bold text indicates improvements in the results, the underlined text indicates the second-best results, and the italics text signifies the worst value.

Attack	Dataset	DT	ADP	Mixup	AdvT	ScRNN	SHIELD	Our method
TextFloor	MR	0.09	0.11	0.13	0.10	*0.02*	0.51	**0.79**
	HS	0.25	0.33	0.46	0.40	*0.15*	0.53	**0.68**
	CB	*0.35*	0.50	0.38	0.39	0.40	0.78	**0.85**
TextBugger	MR	0.15	0.22	*0.11*	0.20	*0.11*	0.43	**0.64**
	HS	*0.33*	0.36	*0.33*	0.54	0.30	0.46	**0.65**
	CB	*0.45*	0.52	0.63	0.55	0.62	**0.77**	0.74
DeepWordBug	MR	0.18	0.11	*0.05*	0.13	0.09	0.61	**0.92**
	HS	0.58	0.31	0.39	0.45	*0.25*	0.73	**0.82**
	CB	0.54	0.49	0.55	0.47	*0.36*	0.74	**0.85**
PWWS	MR	0.17	0.14	*0.12*	0.3	0.13	0.45	**0.58**
	HS	0.27	0.35	0.55	0.40	*0.19*	0.58	**0.60**
	CB	0.45	0.49	0.47	0.60	*0.43*	0.73	**0.80**
AVG		0.385	0.327	0.347	0.344	*0.254*	0.61	**0.74**

**Figure 12:**
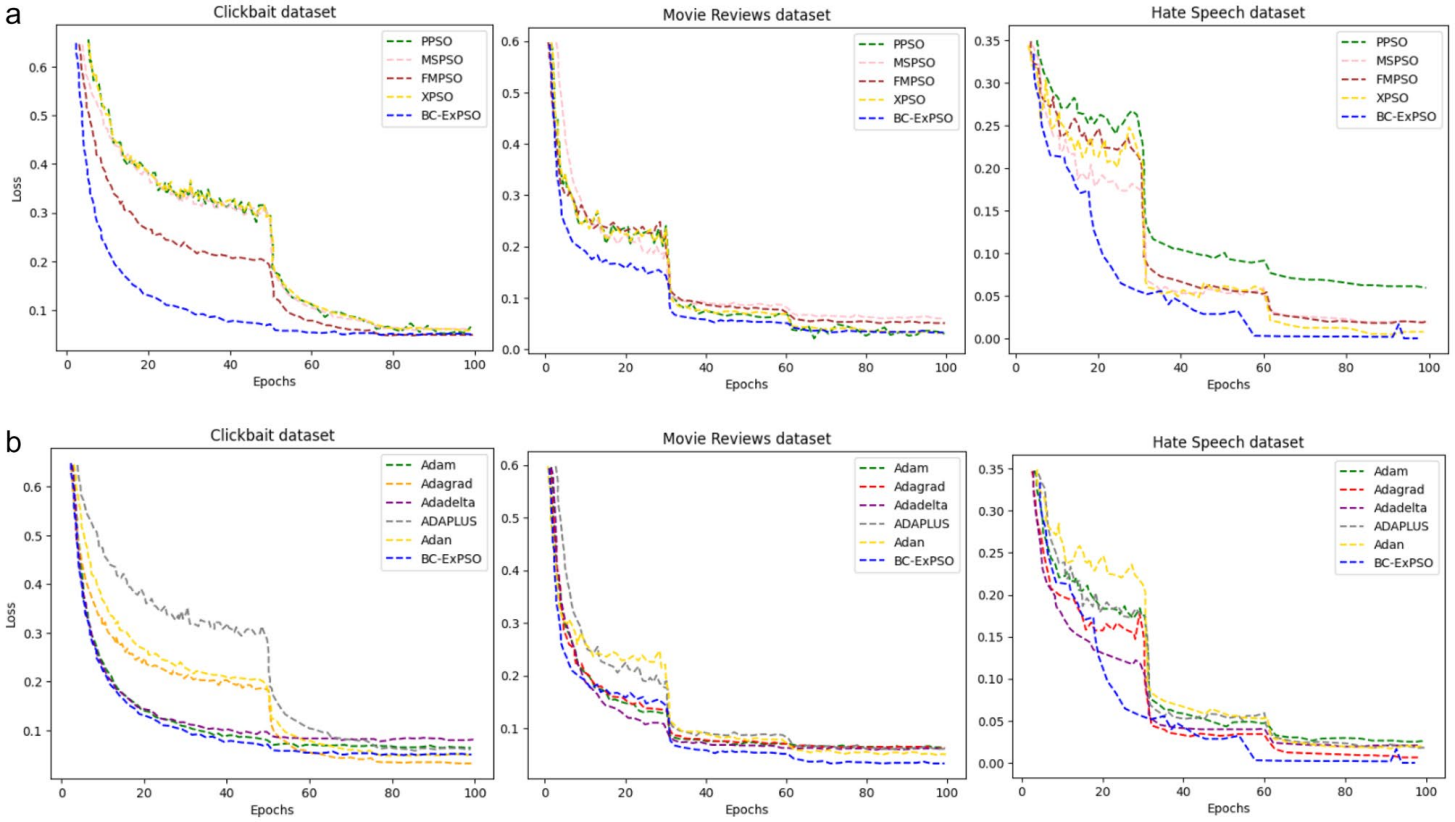
(**a**) Loss comparison during training for the HS, CB, and MR datasets: BC-ExPSO vs. baseline optimizers: PPSO, MSPSO, FMPSO, and XPSO. (**b**) Loss comparison results during training for the HS, CB, and MR datasets: BC-ExPSO vs. baseline optimizers: ADAM, ADAGRAD, ADADELTA, ADAPLUS, and ADAN.

To gauge the effectiveness of our optimization method, we chose to benchmark it against other widely used optimizers for DNNs across three datasets, as illustrated in Fig. 12a,b. While comparing our approach with various optimization techniques based on Particle Swarm Optimization, XPSO, PPSO, FMPSO, and MSPSO begin training with the highest loss for the Clickbait, Hate Speech, and Movie reviewer datasets. Conversely, BC-ExPSO embarked on training with a low initial training loss and consistently achieved the lowest loss by the end of training. Notably, BC-ExPSO demonstrated significantly faster convergence than did the other optimization methods across the HS dataset. BC-ExPSO consistently outperformed the others in terms of loss and convergence from the very beginning of training until the 60th–75th epochs in both the Clickbait and Movie Reviews datasets. After 75 epochs, the BC-ExPSO loss in the MR and CB datasets approached a level similar to that of other optimizers, such as FMPSO, XPSO, and PPSO.

When comparing our optimization approach with the Adam techniques across various datasets, BC-ExPSO stands out for its notably swift convergence in the CB and MR datasets. It consistently achieved the lowest loss after training, particularly in the case of the Hate Speech and Movie Reviews datasets. However, it is worth mentioning that in the CB dataset, the Adagrad optimizer achieved the lowest loss. In summary, in most cases, BC-ExPSO is the superior optimization algorithm in this comparison. Not only does it exhibit faster convergence, but it also attains a significantly lower loss.

Table 9 displays the Friedman and Wilcoxon signed-rank test results with a significance level of 5%. Examining the Friedman mean rank, ExPSO is the top among the approaches, outperforming the other algorithms across diverse benchmark problems. As the Wilcoxon signed-rank test indicates, the results consistently show that ExPSO significantly outperforms the other methods (with no p-values exceeding 0.05). This underscores the power of ExPSO and its ability to strike a proper balance to explore and exploit the search space thoroughly. The comparisons and statistical findings underscore the significant contribution of the exponential search strategy, affirming the superiority of ExPSO in terms of convergence velocity and optimization accuracy.

**Table 9:** Friedmann mean rank and Wilcoxon signed test results with 5% significance.

Average rank	Algorithm	Friedman mean rank	p-value	z-value
			Wilcoxon signed test	
1	BC-ExPSO	4.44	–	–
4	FMPSO	8.38	0.004682	− 2.828147
3	PPSO	6.64	0.000068	− 3.984589
5	XPSO	8.55	0.000044	− 4.084250
2	MSPSO	6.58	0.014889	− 2.435076

**Figure 13:**
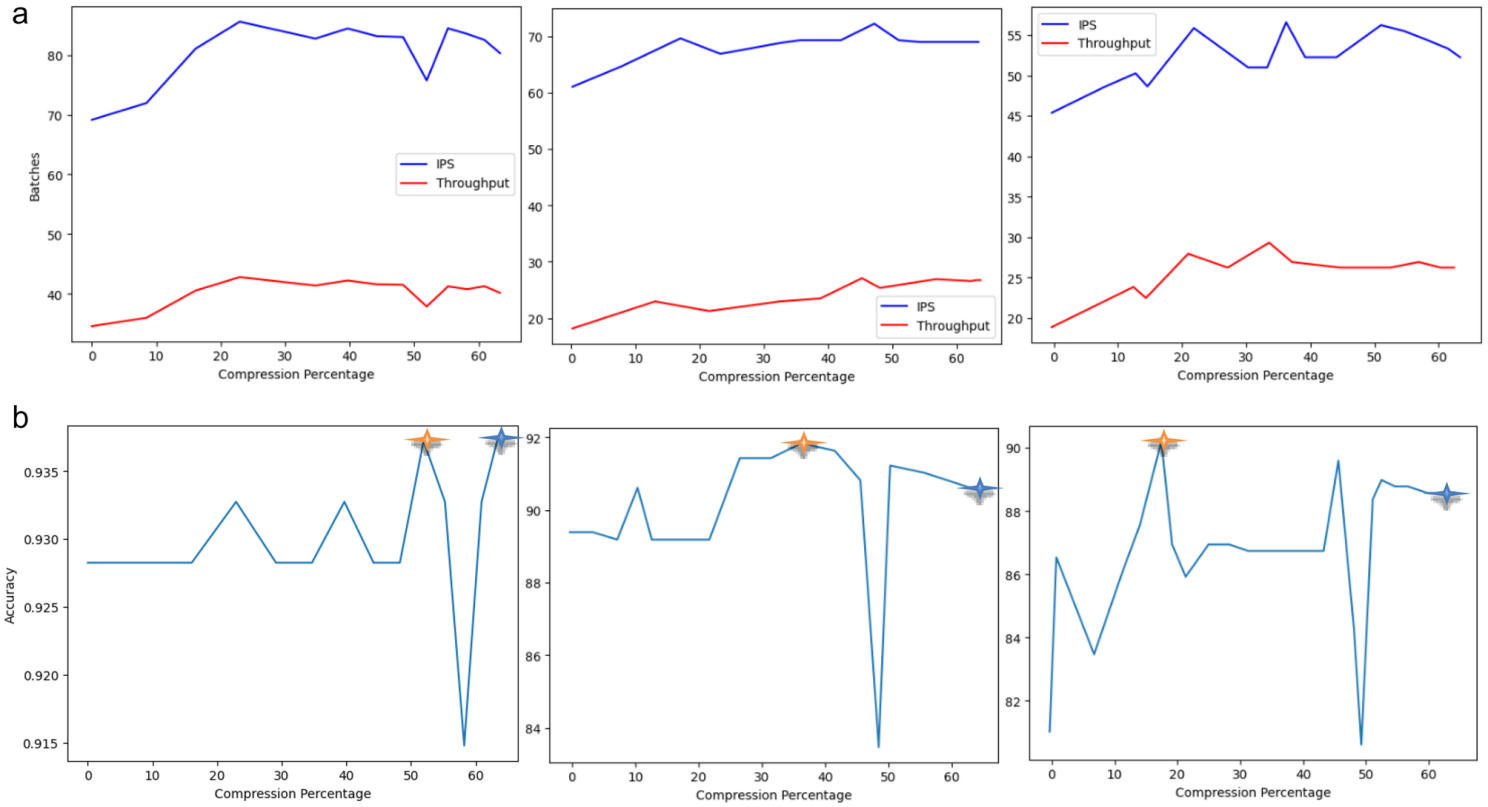
(**a**) Evaluation of our model's performance (throughput and IPS vs. batch size) on the Clickbait, Movie Reviews, and Hate Speech datasets. (**b**) Evaluation of our model's performance (accuracy vs. compression percentage) on the Clickbait, Movie Reviews, and Hate Speech datasets. The gold and blue stars emphasize the optimal compression percentage and the highest compression percentage, respectively, while maintaining accuracy.

In optimizing model performance, achieving faster inference times is a critical objective. To attain this, we explore the changes in the inference per second (IPS), throughput, and accuracy values in Fig. 13. This exploration involved applying various compression percentages to the original model, starting from 0%. Our approach consistently showcases a substantial improvement in Throughput and Inferences Per Second values (Fig. 13a) synchronized with accuracy values (Fig. 13b) as the compression iterations progress across different datasets. We emphasize that our primary goal is to maintain the model's performance throughout the compression process, and this goal is notably achieved, particularly for weights up to 65% on the CB, MR, and HS datasets. Furthermore, it is important to highlight that in certain instances, our approach not only maintains the model's performance but also enhances it. The illustration reveals that specific compression phases may temporarily decrease the model's accuracy, throughput, and IPS. This phenomenon can be attributed to the compression technique, which selectively prunes connections up to certain nodes rather than removing them entirely. Consequently, evaluating the model with the current set of weights may involve redundant connections and nodes, potentially disrupting the decision-making process. In the subsequent compression iterations, ExPSO involves retraining the model with newly updated positions and retaining only the best global parameters. This strategic retraining process ensures that the model discards unusual connections and nodes, significantly improving the accuracy.

Table 10 shows a comparative analysis of our compression approach with other baseline methods on the CB, MR, and HS datasets (executed on a GPU). Our approach emerges as the standout performer with the lowest perplexity (14.28) and the highest model accuracy (93.72%) for the Clickbait and Hate Speech datasets among the four evaluated methods. This finding suggests that we can effectively compress the language model while maintaining superior predictive capabilities. SparseGPT demonstrates impressive performance, closely tracking our method with a perplexity of 16.14 and an accuracy of 92.65% on the HS and CB datasets. Notably, our method outperforms SparseGPT in terms of perplexity on the HS dataset, while SparseGPT excels in terms of accuracy. The decrease in accuracy can be ascribed to the well-known trade-off between compression and accuracy in NLP models. To demonstrate this, we reduced the compression rate of our model from 65 to 50%, resulting in a noticeable enhancement in accuracy (97.65%). This approach brought the performance close to what SparseGPT achieved. Finding the ideal compression percentage that consistently yields optimal results across all evaluation datasets and metrics is challenging.

**Table 10:** Comparison of the perplexity and accuracy of our compression method with those of QuantGPT, KnGPT2, LightPAFF, and SparseGPT.

Dataset	CB		MR		HS	
Compression method	PPL (↓)	Accuracy ($\uparrow$)	PPL (↓)	Accuracy ($\uparrow$)	PPL (↓)	Accuracy ($\uparrow$)
QuantGPT ^17^	45.58	88.13	55.25	80.07	45.93	78.85
KnGPT2 ^18^	23.28	89.25	53.73	81.27	56.8	75.41
LightPAFF ^19^	20.50	92.18	38.78	85.17	37.50	85.47
SparseGPT^57^	16.14	92.65	23.88	**98.13**	30.43	88.36
Our method	**14.28**	**93,72**	**20.14**	91,86	**26.84**	**90.13**

Significant values are in bold.

Table 11 displays the inference latency results of our compression technique vs GPT-2. In achieving a compression rate of 65%, our approach showcases an impressive acceleration, yielding over 6 × speedup on a GPU and an 8 × speedup on a CPU compared to the baseline GPT-2 model. These findings underscore the effectiveness of our method in significantly accelerating inference speeds for GPT-2.

**Table 11:** Comparison of inference speedup for our compression method on GPT-2. Evaluation is conducted by generating one sentence at a time autoregressively.

Method	Compression percentage	Number of parameters	(GPU)		(CPU)	
			Latency	Speedup	Latency	Speedup
GPT-2	0%	345 M	553 ms	1.00×	1,683 ms	1.00×
Our method	10%	280 M	440 ms	1.25×	1,255 ms	1.34×
	20%	19 M	307 ms	1.80×	868 ms	1.93×
	30%	80 M	234 ms	2.36×	582 ms	2.89×
	40%	72 M	167 ms	3.31×	457 ms	3.68×
	50%	62 M	132 ms	4.18×	367 ms	4.58×
	**65%**	**60 M**	**90 ms**	**6.14×**	**200 ms**	**8.41×**

Significant values are in bold.

### Discussion

In our methodology, stochasticity arises from three components: (i) the inherent randomness in the outputs of base models, (ii) the assignment of the main prediction head, and (iii) the variability in the Gumbel–Softmax outputs. Concerning point (i), despite the nondeterministic nature of the outputs from multi-version training base models, the relative ranking of inputs for the expert predictor remains consistent during each inference call. This consistency is achieved through minor changes in representation, justified by training on randomly different portions of the same data. Importantly, these adjustments resulted in high-performing outcomes without compromising the fidelity of the model across various runs (Fig. 11). (ii) Occurs in a typical iterative black-box process, where an attacker experiments with various manipulations of a given text. As the attacker manipulates these, the input text to the model changes at each iterative step. Consequently, the assignment of heads also changes because each prediction head specializes in different features, such as specific words or phrases in an input sentence. Therefore, for a given input, the assignment of expert predictors remains consistent for a particular set of manipulations. Consequently, no additional information is gleaned even if an attacker repeatedly submits the model with specific changes to the original sentence. Regarding (iii), despite the nondeterministic nature of Gumbel-Softmax outputs, the relative ranking of the expert predictor is consistently preserved during each inference call by maintaining a sufficiently small value for τ. This approach ensures that the variability introduced by Gumbel-Softmax does not compromise the model's fidelity across different runs (Tables 6, 7, and 8).

To evaluate the importance of each stochastic method on the overall effectiveness of the model, we test the basic GPT-2 model with only the stochastic multi-expert (SME) or multi-version compressed neural network training (MVC-NNT) modules. Table 12 shows that (GPT-2 + SME) and (GPT-2 + MVC-NNT) perform differently across different datasets and attacks. Specifically, we observe that MVC-NNT performs better than the SME module in the case of the MR dataset, SME is better than the MVC-NNT module in the case of the HS dataset, and we have mixed results in the CB dataset. Nevertheless, the final model, which comprises both the SME and MVC-NNT modules, consistently performs the best across all the cases. This shows that both the SME and MVC-NNT modules complement each other and are crucial for the final model's robustness.

**Table 12:** Complementary role of SME or MVC-NNT under TextFloor (TF), TextBugger (TB), DeepWordBug (DW), and Probability Weighted Word Saliency (PS) attacks.

Dataset	MR				HS				CB			
Attack	TF	TB	DW	PS	TF	TB	DW	PS	TF	TB	DW	PS
Basic model	0.02	0.10	0.08	0.13	0.13	0.30	0.27	0.19	0.36	0.62	0.31	0.50
Only SME	0.32	0.30	0.17	0.28	0.57	0.50	0.36	0.42	0.35	0.43	0.52	0.64
Only MVC-NNT	0.65	0.39	0.64	0.35	0.27	0.24	0.25	0.42	0.67	0.59	0.32	0.55
Final Model	**0.79**	**0.64**	**0.92**	**0.58**	**0.68**	**0.65**	**0.82**	**0.60**	**0.85**	**0.74**	**0.85**	**0.80**

Significant values are in bold and underline.

Most previous works in adversarial defense have been designed for a specific type of word, synonym substitution, as in certified training, or misspelling level of attack. Thus, these algorithms are usually evaluated against a small subset of (≤ 4) attack methods. Although some works propose general defense methods, they are often built upon adversarial training, which requires training everything from scratch^38,75–77^. In contrast to previous approaches, our methodology does not address the characteristics of the resulting perturbations from attackers but rather addresses their fundamental attack mechanism, which is usually an iterative perturbation optimization process (Fig. 1). This allows our approach to effectively defend against 14 different black-box attacks (Tables 7 and 8), demonstrating its effectiveness in practice. Furthermore, our method enables us to “hot-fix” a complex NN by replacing and training only the last layer, removing the necessity of retraining the entire model from scratch.

Additionally, previous methods (e.g., DT and ADP) mainly aim to reduce the dimensionality of the adversarial subspace, i.e., the subspace containing all adversarial examples, by forcing adversaries to attack a single fixed ensemble of diverse sub-models simultaneously. This helps to improve the transferability of robustness to different tasks. However, our approach primarily focuses on diluting direct attacks rather than transferring them. We achieve this by compelling adversaries to target stochastic, different meanings, and ensemble sub-model variations during each inference pass. Additionally, we increase the model's generalization capabilities by utilizing the average expert architectures from each head; this decision mitigates the potential risk of the model becoming excessively specialized, which could otherwise limit its capacity to discover novel insights, as exemplified by the SHIELD approach, which selects only the single best-performing architecture. Moreover, our approach leverages training on diverse dataset partitions and the utilization of various compression rates. This diversity in training perspectives empowers the model to encompass a more extensive spectrum of patterns and features, bolstering its robustness against adversarial attacks. In contrast, Mixup, AdvT, and ScRNN employ relatively straightforward training processes that sometimes do not sufficiently explore the full range of potential adversarial perturbations or attack strategies^39^.

Some compression techniques are specific to particular PLM architectures. For example, the KnGPT2 technique mentioned in ^18^ was designed for GPT-2. Applying this approach to other GPT versions or models might not yield the same benefits. As a result, our compression technique can be applied to both encoder and decoder-based pre-trained language models, independent of the model's internal architecture. Moreover, careful parameter pruning and management can significantly reduce the number of model parameters while maintaining high accuracy (Figs. 12, 13, and Table 10). This approach mitigates memory-intensive demands and requires minimal additional computational resources during the compression process due to batch-generative exponential particle swarm optimization. This stands in contrast to methods such as SparseGPT, which still rely on substantial computational resources for compression.

### Limitations and future work

Our compression technique exhibits agnosticism, extending its application to various neural network architectures, including CNNs, RNNs, and transformer-based models^78^. It makes it adaptable for tasks and domains, such as question-answering and language generation^79^.

In this research, we restrict the architecture of each expert to a fully connected layer with a maximum of three hidden layers (corresponding to the number of experts). While recognizing the potential for enhanced diversity in submodels^80^, exploring a wider array of model architectures encompassing various versions of compressed GPTs with diverse attention layers might be advantageous^81^. Nonetheless, it is crucial to note that such exploration may involve trade-offs, particularly in computational time.

Recent experiments have revealed that scheduled unfreezing methods can narrow the performance gap with full fine-tuning, achieving state-of-the-art transfer performance. This finding suggested that such methods extend beyond merely mitigating catastrophic forgetting^82^. Although our primary focus is not on robust transferability, our approach can readily accommodate it by simply unfreezing the base layers, denoted as $f\left(x,{ \theta }_{L-1}^{*}\right)$.

Our compression technique is constrained by an initial fixed number of iterations, potentially leading to premature termination before the optimal positions are reached (global best positions) or training is extended until the maximum number of iterations, which may incur unnecessary computational costs. If we propose an early stopping method that considers the accuracy history values in the compression process, the adaptive approach ensures that our compression technique halts training when meaningful convergence or performance improvement is observed, leading to overcoming the limitation of a predefined max iteration.

## Conclusion

In our research paper, we presented a comprehensive evaluation of innovative methods designed to optimize defense models' performance, efficiency, and robustness. We conducted this assessment using three publicly available datasets. Our evaluation framework encompasses various metrics, including weighted F1 scores, performance in the face of adversarial attacks, computational efficiency, and language model perplexity. The results of our approaches were promising across different scenarios and underscored their potential for real-world applications that prioritize data integrity, efficiency, and security. This approach has successfully achieved stability and robustness by mitigating the risk of overfitting to specific batch characteristics through weight compression. This approach reduces memory requirements and streamlines predictions, making it suitable for real-time applications while contributing to energy savings. The statistical results (Wilcoxon signed rank and Friedman rank) show that the exponential search strategy significantly contributes to the search process and proves the superiority of the compression optimization method in terms of convergence velocity and optimization accuracy. Moreover, compression makes it more challenging for adversaries to reverse-engineer the model architecture or extract sensitive information. Furthermore, our strategy, involving the creation of an ensemble of experts with stochastic weights, a random selection of compressed-based models, and training across different segments of datasets, enhances the model's resistance to adversarial attacks. This is primarily because attackers find it increasingly difficult to predict how the model combines its predictions, bolstering its security. In our future research endeavors, we intend to expand the applicability of our compression approach. This expansion will involve conducting experiments with diverse pre-trained models spanning various domains.

## Data Availability

The datasets used during the current study are available in the GitHub repositories: Clickbait dataset (CB) in https://github.com/ankeshanand/deep-clickbait-detection, Hate Speech dataset (HS) in https://github.com/t-davidson/hate-speech-and-offensive-language, and Movie Reviews dataset (MR) in https://github.com/shekhargulati/sentiment-analysis-python/tree/master/polarity-data/rt-polaritydata.
